# Epigenetic clock and methylation studies in marsupials: opossums, Tasmanian devils, kangaroos, and wallabies

**DOI:** 10.1007/s11357-022-00569-5

**Published:** 2022-04-21

**Authors:** Steve Horvath, Amin Haghani, Joseph A. Zoller, Ken Raj, Ishani Sinha, Todd R. Robeck, Pete Black, Aidan Couzens, Clive Lau, Meghety Manoyan, Yadiamaris Aviles Ruiz, Annais Talbott, Katherine Belov, Carolyn J. Hogg, Karen E. Sears

**Affiliations:** 1 Human Genetics, David Geffen School of Medicine, University of California, Los Angeles, CA 90095 USA; 2 Department of Biostatistics, Fielding School of Public Health, University of California, Los Angeles, Los Angeles, CA USA; 3 Radiation Effects Department, Centre for Radiation, Chemical and Environmental Hazards, Public Health England, Chilton, Didcot, UK; 4 Department of Ecology and Evolutionary Biology, University of California at Los Angeles, Los Angeles, CA 90095 USA; 5 Zoological Operations, SeaWorld Parks and Entertainment, 8008 SeaWorld Drive, Orlando, FL USA; 6 Busch Gardens Tampa, Tampa, FL USA; 7 Ciencias Naturales, Universidad Interamericana de Puerto Rico, Bayamón, 00958 Puerto Rico; 8 School of Life and Environmental Sciences, The University of Sydney, Sydney, NSW Australia

**Keywords:** Opossum, Aging, Development, Epigenetic clock, DNA methylation

## Abstract

The opossum (*Monodelphis domestica*), with its sequenced genome, ease of laboratory care and experimental manipulation, and unique biology, is the most used laboratory marsupial. Using the mammalian methylation array, we generated DNA methylation data from *n* = 100 opossum samples from the ear, liver, and tail. We contrasted postnatal development and later aging effects in the opossum methylome with those in mouse (*Mus musculus*, C57BL/6 J strain) and other marsupial species such as Tasmanian devil, kangaroos, and wallabies. While the opossum methylome is similar to that of mouse during postnatal development, it is distinct from that shared by other mammals when it comes to the age-related gain of methylation at target sites of polycomb repressive complex 2. Our immunohistochemical staining results provide additional support for the hypothesis that PRC2 activity increases with later aging in mouse tissues but remains constant in opossum tissues. We present several epigenetic clocks for opossums that are distinguished by their compatibility with tissue type (pan-tissue and blood clock) and species (opossum and human). Two dual-species human-opossum pan-tissue clocks accurately measure chronological age and relative age, respectively. The human-opossum epigenetic clocks are expected to provide a significant boost to the attractiveness of opossum as a biological model. Additional epigenetic clocks for Tasmanian devil, red kangaroos and other species of the genus *Macropus* may aid species conservation efforts.

**Supplementary Information:**

The online version contains supplementary material available at 10.1007/s11357-022-00569-5.

## Introduction

*Monodelphis domestica* (the gray short-tailed opossum) is a South American marsupial that is used as a mammalian model system [1–3] in countries including the USA, the UK, Canada, Germany, Poland, Italy, Japan, Brazil, and Australia. Opossums are used as model systems because of their suitability to lab use and the advantages presented by their unique biology relative to placental mammals such as mice. Regarding the former, opossums are small, relatively docile, amenable to laboratory rearing, and have had their genome sequenced [1, 2, 4, 5]. Regarding the latter, opossums are born after only 14–15 days gestation at a developmental stage equivalent to an E10.5–12.5 mouse or a human at 40 days of gestation [1, 6]. Because of their early birth, much of opossum development happens outside the uterus, and can be studied and manipulated more easily than in traditional mammalian models such as mice [1]. This is especially the case as gray short-tailed opossums, although marsupials, lack a pouch. Researchers have taken advantage of these traits to study development of the lung [7], neural crest [8], brain [9–11], limb [12, 13], reproductive [14, 15], and other systems. Because of their early developmental state at birth, newborn opossums are also capable of totally regenerating their spinal cord even after it has been completely severed, and therefore provide an ideal model for spinal cord regeneration [9, 16–18]. Opossums are also one of the only mammals besides humans that develop UV-induced melanoma, making them a good model for melanoma research [19–21]. Opossums are also being used to study many other biomedically-relevant topics, including diet-induced hyperlipidemia [22, 23], wound healing [24], teratogenesis [25, 26], and susceptibility to infection [27, 28]. Another aspect of marsupial biology that differs from that of placentals is their longevity; marsupials, on average, live only about 80% as long as similarly-sized, non-flying placentals [29]. Beyond biomedical topics, opossums have been used to study a suite of evolutionary topics, most of which have compared evolution in marsupial and placental mammals [30, 31].

DNA methylation-based age estimators have been developed for many mammalian species but not yet for opossums [32–39]. Here, we present highly accurate epigenetic clocks that apply to both humans and opossum. The human-opossum clock increases the probability that findings in opossums will translate to humans, and vice versa. Since opossums are already being used in biomedical research, we expect that these clocks will facilitate research into development and the influence of age on pathology. We also contrast the methylomes of opossums and mice during postnatal development and later aging, and across later aging in additional marsupial species. This approach has the potential to yield important insights into development, aging, and the relationship between these processes across mammals, as well as provide a critical foundation for future, experimental research on these important topics.

## Methods

### Opossum and mouse tissue samples

Opossum samples come from a pedigreed, breeding colony of gray short-tailed opossums (*Monodelphis domestica*) that was established by founder individuals purchased from the Southwest Foundation for Biomedical Research. Mouse samples come from a breeding colony of C57BL/6 J mice originally purchased from the Jackson Laboratory. Both colonies are maintained by the Sears Lab at UCLA. Opossums were euthanized by CO_2_ inhalation to effect followed by bilateral thoracotomy. Mice were euthanized by CO_2_ inhalation to effect followed by cervical dislocation. These procedures are in accordance with the AVMA Guidelines for the Euthanasia of Animals 2013: https://www.avma.org/KB/Policies/Documents/euthanasia.pdf, and all animal procedures were approved by the UCLA IACUC. All tissue samples, e.g., liver, blood, tail (see Table 1), were taken from euthanized opossums and mice and stored at -20° C until use. DNA from samples was extracted and purified using a DNA Miniprep Plus Kit (Zymo), following manufacturer’s protocols. PicoGreen fluorescent dsDNA was used to assess the concentration of resulting DNA and concentrations adjusted to 50 to 250 ng/ul. DNA samples were submitted to the Technology Center for Genomics & Bioinformatics at UCLA for generation of DNA methylation data and further analyses. Our EWAS of age in liver samples from Mus musculus used *N* = 498 mouse liver samples from the Mammalian Methylation Consortium.

**Table 1:** Description of marsupial and mouse methylation data. Tissue type, *N* = total number of samples/arrays. Number of females. Age: mean, minimum and maximum in units of years

Species	Tissue	N	No. Female	Mean Age (Years)	Min. Age	Max. Age
Opossum	Ear	45	28	0.771	0.0384	2.22
	Liver	48	26	0.922	0.0384	3.27
	Tail	7	4	0.164	0.0384	0.288
Eastern grey kangaroo	Blood	12	5	4.517	1.820	13.300
Red kangaroo	Blood	37	20	7.097	0.620	11.730
Red-necked wallaby	Blood	5	4	7.584	2.020	9.650
Tasmanian devil	Ear	41	23	3.427	0.500	8.000
Western grey kangaroo	Blood	5	3	2.708	1.666	5.036
Mouse	Blood	97	1	0.998	0.0192	2.250
	Ear	18	1	0.0671	0.0192	0.115
	Liver	498	1	0.0367	0.0191	2.778
	Muscle	18	1	0.0671	0.0192	0.115
	Tail	18	1	0.0671	0.0192	0.115
	Whole Brain	18	1	0.0671	0.0192	0.115

### Tasmanian devil tissue samples

Ear samples from Tasmanian devil (*Sarcophilus harrisii*) were collected from individuals in the Tasmanian devil insurance metapopulation, either living as an introduced population on Maria Island (*N* = 29), or in the zoo-based population (*N* = 17). Samples were collected by the Save the Tasmanian Devil Program (STDP), or the respective zoos, under the STDP’s *Standard Operating Procedure: Trapping and handling wild Tasmanian devils* and shared with the University of Sydney. The samples were collected for standard management practice, which received ethics approval over 15 years ago. DNA from these samples were made available for use in this study, no individuals were specifically sampled for the purposes of this research.

Tasmanian devils within the zoo-based insurance population are housed in a range of scenarios from intensive housing as individuals, or as pairs in breeding season; or in large group housed enclosures, ranging from 4–10 males and 4–10 females per enclosure [40]. Tasmanian devils are not known to be a social species and housing within the Tasmanian devil insurance population is under the management of the Zoo and Aquarium Association Australasia [40]. Devils are housed and fed according a variety of diet items and managed according to the ZAA Husbandry Guidelines for the species [41]. Devils living on Maria Island are wild devils and so there is no animal care or maintenance for these individuals other than twice yearly monitoring trapping trips.

Samples were selected for this study to include a range of known age individuals and sexes. DNA was extracted using either a modified phenol–chloroform protocol [42] or the MagAttract HMW DNA kit (Qiagen, Germany; cat: 67,563). DNA concentration and quality were assessed using a Nanodrop 2000 Spectrophotometer (ThermoFisher Scientific) and 0.8% agarose gel electrophoresis for 30 min at 90 V.

### Kangaroo and wallaby tissue samples

The blood samples for kangaroo and wallaby were opportunistically collected from zoo-based animals during routine health exams. As detailed in Table 1, we analyzed blood from the following species: *Macropus rufus* (red kangaroo), *Macropus giganteus* (Eastern grey kangaroo), *Macropus fuliginosus* (Western grey kangaroo), and *Macropus rufogriseus* (red-necked wallaby).

### Human tissue samples

To build the human-opossum clock, we analyzed previously generated methylation data from *n* = 1366 human tissue samples (adipose, blood, bone marrow, dermis, epidermis, heart, keratinocytes, fibroblasts, kidney, liver, lung, lymph node, muscle, pituitary, skin, and spleen) from human individuals whose ages ranged from 0 to 93. The original tissue samples came from several sources [43]. Tissue and organ samples came from the National NeuroAIDS Tissue Consortium [44, 45]. Blood samples were obtained from PEG and the Cape Town Adolescent Antiretroviral Cohort study [46]. Skin and other primary cells were provided by Kenneth Raj [47]. Ethics approval (IRB#15–001,454, IRB#16–000,471, IRB#18–000,315, IRB#16–002,028).

### DNA methylation data

To overcome the species barrier, we used a DNA methylation array platform (HorvathMammalMethylChip40) that encompasses CpGs flanked by DNA sequences that are conserved across different species of the mammalian class [48]. All methylation data were generated using the mammalian array platform. Not all CpGs on the array apply to opossums. The particular subset of species for each probe is provided in the chip manifest file at the NCBI Gene Expression Omnibus (GEO) platform (GPL28271). The SeSaMe normalization method was used to define beta values for each probe [49].

### Relative age estimation

To introduce biological meaning into age estimates of opossums and humans, two species with very different lifespans; as well as overcome the inevitable skewing due to unequal distribution of data points from opossums and humans across age range, relative age estimation was made using the formula: Relative age = Age/maxLifespan where the maximum lifespan for the two species was chosen from an updated version of the *anAge* data base [50]. According to the “anAge” data base [50, 51], the maximum lifespan of Monodelphis domestica is 4.2 years.

### Clocks and penalized regression

Details on the clocks (CpGs, genome coordinates) and R software code are provided in the Supplement. Penalized regression models were created with glmnet [52]. We investigated models produced by “elastic net” regression (alpha = 0.5 in glmnet R function). The optimal penalty parameters in all cases were determined automatically by using a tenfold internal cross-validation (cv.glmnet) on the training set. By definition, the alpha value for the elastic net regression was set to 0.5 (midpoint between Ridge and Lasso type regression) and was not optimized for model performance. We performed a cross-validation scheme for arriving at unbiased (or at least less biased) estimates of the accuracy of the different DNAm based age estimators. One type consisted of leaving out a single sample (LOOCV) from the regression, predicting an age for that sample, and iterating over all samples. We subset the set of CpG probes to those that uniquely mapped to a CpG site in the respective species. While no transformation was used for the blood clock for opossums, we did use a log linear transformation for the dual species clock of chronological age (also known as calendar age), as detailed in the Supplement. The accuracy of the resulting clocks was assessed via cross validation estimates of 1) the correlation R between the predicted epigenetic age and the actual (chronological) age of the animal, 2) the median absolute difference between DNAm age and chronological age (mae).

### GREAT analysis

We analyzed gene set enrichments using GREAT (Version 3, Human hg19) [53]. The GREAT enrichment analysis automatically conditioned out (removed) any bias resulting from the design of the mammalian array by using a background set of CpGs that map to opossums and are located on the mammalian array. The GREAT software performs both a binomial test (over genomic regions) and a hypergeometric test over genes.

We performed the enrichment based on default settings (rule: basal plus extension, 5.0 kb upstream, 1.0 kb downstream, Distal: advanced span 50 kb) for gene sets implemented in GREAT. To avoid large numbers of multiple comparisons, we restricted the analysis to the gene sets with between 10 and 3,000 genes. We report nominal P values and two adjustments for multiple comparisons: Bonferroni correction and the Benjamini–Hochberg false discovery rate.

### EWAS and functional enrichment

EWAS was performed in each tissue separately using the R function “standardScreeningNumericTrait” from the “WGCNA” R package [54]. The resulting data were combined across tissues using Stouffer's meta-analysis method. The functional enrichment analysis was done using the genomic region of enrichment annotation tool [53]. CpGs implicated by our EWAS were filtered for CpG position information, lifted over to the human genome using UCSC’s Liftover tool and fed into the online functional analysis tool GREAT using the default mode, to obtain a list of significantly enriched functions for both positive and negative EWAS hits in the different tissues. We used a hypergeometric test to characterize the chromatin state of age related CpGs [55].

### Immunohistochemical analysis

To further interrogate differences in PRC2 activity suggested by the methylation profiles, we used immunohistochemistry (IHC) to visualize Ezh2 localization in the livers of younger and older adult opossums (9 months and 3 years; from the Sears Lab colony) and mice (12 and 84 weeks; C57BL/6 J mice from Jackson Lab). For reference, C57BL/6 J mice are considered young adults at ~ 12–24 weeks, middle-aged at ~ 40–56, and old at 72 + [56]. Based on our 10 + years of experience with our opossum colony and the life history of the species, we consider 9-month-old opossums to be young adults and 3-year-olds to be older adults [1, 4, 57]. Three individuals were sampled for each timepoint and at least 3 sections stained for each individual. *Ezh2* is the catalytic subunit of the PRC2 and plays a critical role in the complex’s trimethylation of H3K27 to H3K27me3 [58, 59]. Liver tissues were obtained from euthanized opossums and mice, fixed in 4% paraformaldehyde, stored in 100% methanol, and cryosectioned. For IHC, sections were rehydrated through a graded series of methanol to 1 × PBS. 0.3% hydrogen peroxide treatment was used to block exogenous peroxidase activity and heat induced antigen retrieval was carried out by microwaving samples for 15 min in preheated 0.1 M Sodium citrate pH6 buffer. Sections were then exposed to 0.5% triton-x and blocked using a 0.1% triton-x and 10% heat-inactivated goat serum blocking buffer followed by application of the avidin/biotin blocking kit following manufacturer’s instructions (SP-2001, Vector Labs). Sections were then treated overnight at 4 °C with a primary antibody for Ezh2 (Ezh2 [D2C9] XP Rabbit mAB #5246, Cell Signaling; 1:150 dilution). Following repeated 1 × PBS washes, goat anti-rabbit IgG secondary antibody (BA-1000, Vector Labs) was added at a 1:600 dilution in blocking buffer. Secondary antibody was then washed off using 1 × PBS and the Vectastain ABC-HRP Kit, Peroxidase (PK-4000, Vector Labs) applied as per manufacturer’s instructions. Staining was visualized using the DAB Substrate Kit, Peroxidase (SK-4100, Vector Labs), following manufacturer’s instructions and sections were counterstained with hematoxylin, cleared in xylene, and mounted with Permount (Fisher). All sections were imaged at 20 × using a Leica DM IL inverted contrasting microscope. Ezh2 localization was quantified for standardized tissue areas for each section using the threshold tool in ImageJ (imagej.nih.gov) and younger and older animals statistically compared using Mann–Whitney *U*-tests.

## Results

In total, we analyzed *n* = 100 opossum samples from ear, liver, and tail as detailed in Table 1. Our study of tail samples was limited because we only analyzed *N* = 7 tail samples and the maximum age of the animals from which tails were sampled was 0.288 years, which is pre-pubertal. For this reason, we focused on developing a pan tissue clock for opossums that applies to all tissue samples.

As one of our aims was to contrast postnatal development between opossums and mice, we also used the same methylation array platform to also profile *N* = 105 mouse tissue samples (blood, liver, tail, ear, muscle, whole brain) from postnatal weeks 1 to 6 as detailed in Table 1.

Unsupervised hierarchical clustering revealed that the opossum and mouse samples cluster by tissue type (Supplementary Fig. S1, Supplementary Fig. S2). To assess whether platemap errors occurred, we constructed random forest predictors to tissue type and sex in each species. These classifiers exhibit perfect accuracy, with respective (out-of-bag) error rates of zero.

### Predictive accuracy of the epigenetic clock

To arrive at unbiased estimates of the epigenetic clocks, we applied cross-validation analysis with the training data. For the development of the basic opossum clock, this consisted of opossum blood, liver, and tail DNA methylation profiles. For the generation of human-opossum clocks, the training data was constituted by both human and opossum DNA methylation profiles. Cross-validation analysis reports unbiased estimates of the age correlation R (defined as Pearson correlation between the age estimate (DNAm age) and chronological age) as well as the median absolute error.

From these analyses, we developed three epigenetic clocks for opossums that vary with regards to two features: species and measure of age. The pan-tissue opossum clock was trained on 3 tissues (ear, liver, tail) and is expected to generalize to other tissues as well.

**Fig. 1:**
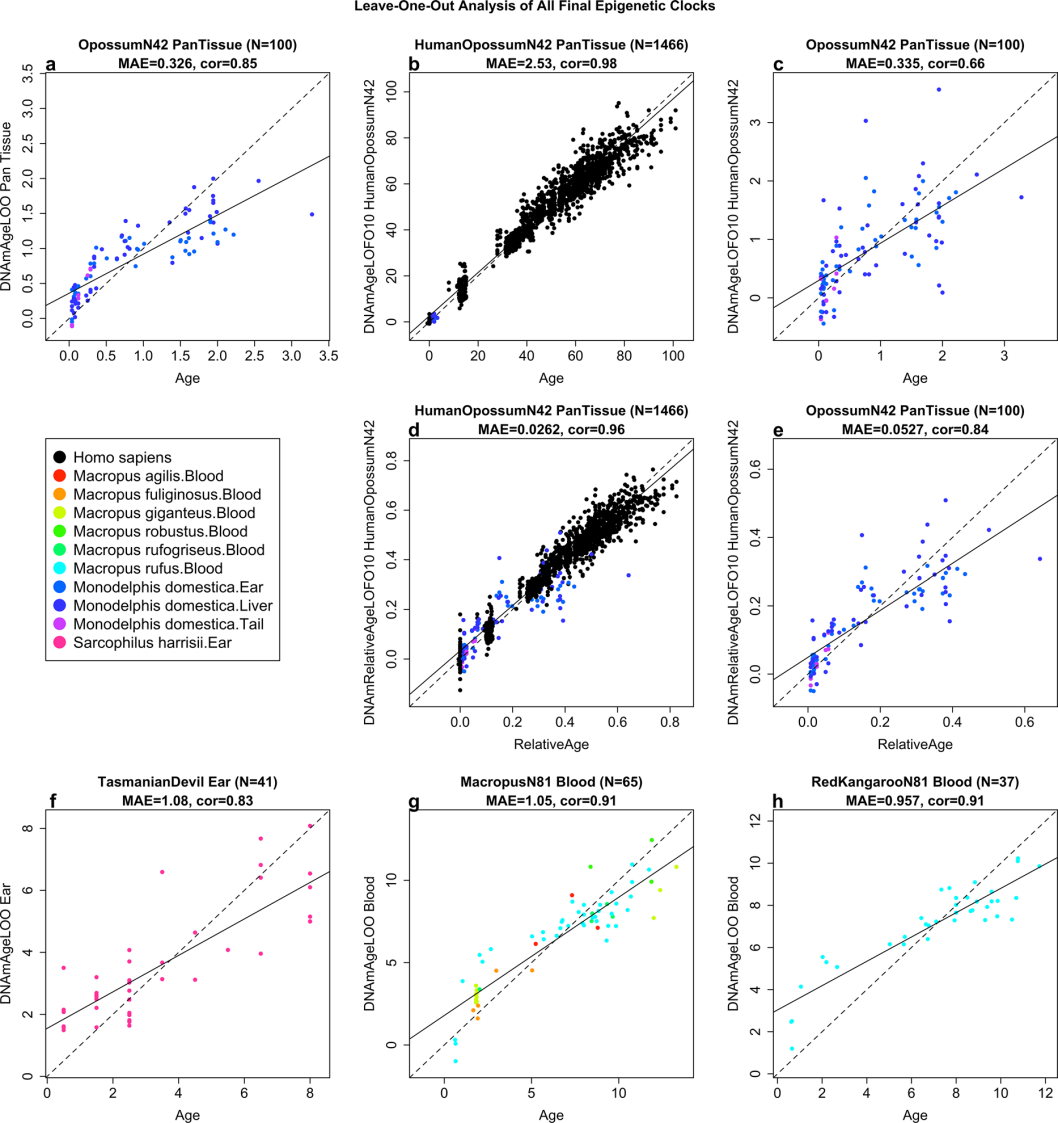
Epigenetic clocks for opossums, Tasmanian devil, red kangaroos, and Macropus. Each panel reports a cross validation estimate of DNA methylation age (y-axis) versus chronological age (in years). We developed 3 epigenetic clocks for opossums: **a**, pan-tissue clock that was trained with opossum tissues only, **b,c**, human-opossum clock for chronological age applied to **b**, both species and **c**, opossums only. Human-opossum clock for relative age applied to **d**, both species and **e,** opossums only. Leave-one-sample-out (LOO) estimate (y-axis, in units of years) versus chronological age or relative age (x-axis). Relative age is defined as chronological age divided by the maximum age of the respective species. **f,** Epigenetic clock for ear samples of Tasmanian devils based on CpGs that map to the genome of *Sarcophilus harrisii*. **g**, Blood clock for species from the genus Macropus. Due to the low sample size, we combined all blood samples from species in the marsupial genus Macropus (i.e., species in the family Macropodidae). Specifically we used blood samples from *Macropus rufus* (red kangaroo), *Macropus giganteus* (Eastern grey kangaroo), *Macropus fuliginosus* (Western grey kangaroo), *Macropus rufogriseus* (red-necked wallaby) as detailed in Table 1. Detailed results for each Macropus species are presented in Fig. S9. **h**, epigenetic clock for red kangaroos. The linear regression of epigenetic age is indicated by a solid line while y = x is depicted by a dashed line. Each title reports the sample size (N), median absolute error, and Pearson correlation coefficient)

The two human-opossum clocks mutually differ by way of age measurement. One estimates chronological ages of opossums and humans (in units of years), while the other estimates relative age, which is the ratio of chronological age of an animal to the maximum lifespan of its species; with resulting values between 0 and 1. We prefer the human opossum clock for relative age because it is more accurate in opossums (*R* = 0.84, Fig. 1e) and arguably more meaningful for biological studies. The relative age estimate facilitates a biologically meaningful comparison between species with very different lifespans such as opossum (4.2 years) and human (122.5 years), which cannot otherwise be afforded by direct comparison of their chronological ages.

As indicated by its name, the pure opossum clock is highly accurate in age estimation of all the tested opossum tissues (*R* = 0.85, and median error = 0.33 years, Fig. 1a). The pan-tissue clocks exhibit high age correlations in individual opossum tissues (Supplementary Fig. S3).

While the pure opossum clock exhibits a very high correlation with age this does not imply high concordance with chronological age: the estimated age of most opossum samples can be considerably different from their chronological age (i.e., the calendar age at the time of sample collection) as reflected by a high median error (defined as median value of the absolute difference between age estimate and actual age).

The human-opossum clock for chronological age leads to high age correlations when DNA methylation profiles of both species are analyzed together (*R* = 0.98, Fig. 1b), but is less accurate when restricted to opossum tissue samples (*R* = 0.66, Fig. 1c). The human-opossum clock for relative age exhibits superior accuracy when restricted to opossums (*R* = 0.84, Fig. 1e). The use of relative age circumvents the clustering of data points of opossums and humans to opposite parts of the curve, which is evident in Fig. 1b. The human-opossum clock of relative age facilitates the comparison between humans and opossums based on their relative positions within the lifespans of both species.

Our sample size was also sufficient to build epigenetic clocks for Tasmanian devils (*N* = 41, Fig. 1f). In contrast, the sample sizes for all but one species from the genus *Macropus* were insufficient for building species specific clocks. After combining the blood samples of these species, we arrived at a sample size (*N* = 65 blood samples) that allowed us to build an epigenetic clock for the *Macropus* genus (*R* = 0.91 Fig. 1g). By construction, this *Macropus* clock is expected to apply to blood samples from all species of this genus. We also present an epigenetic clock for red kangaroos (*Macropus rufus*) that was trained in *N* = 36 blood samples (again *R* = 0.91, Fig. 1h).

### EWAS of chronological age in opossum

**Fig. 2:**
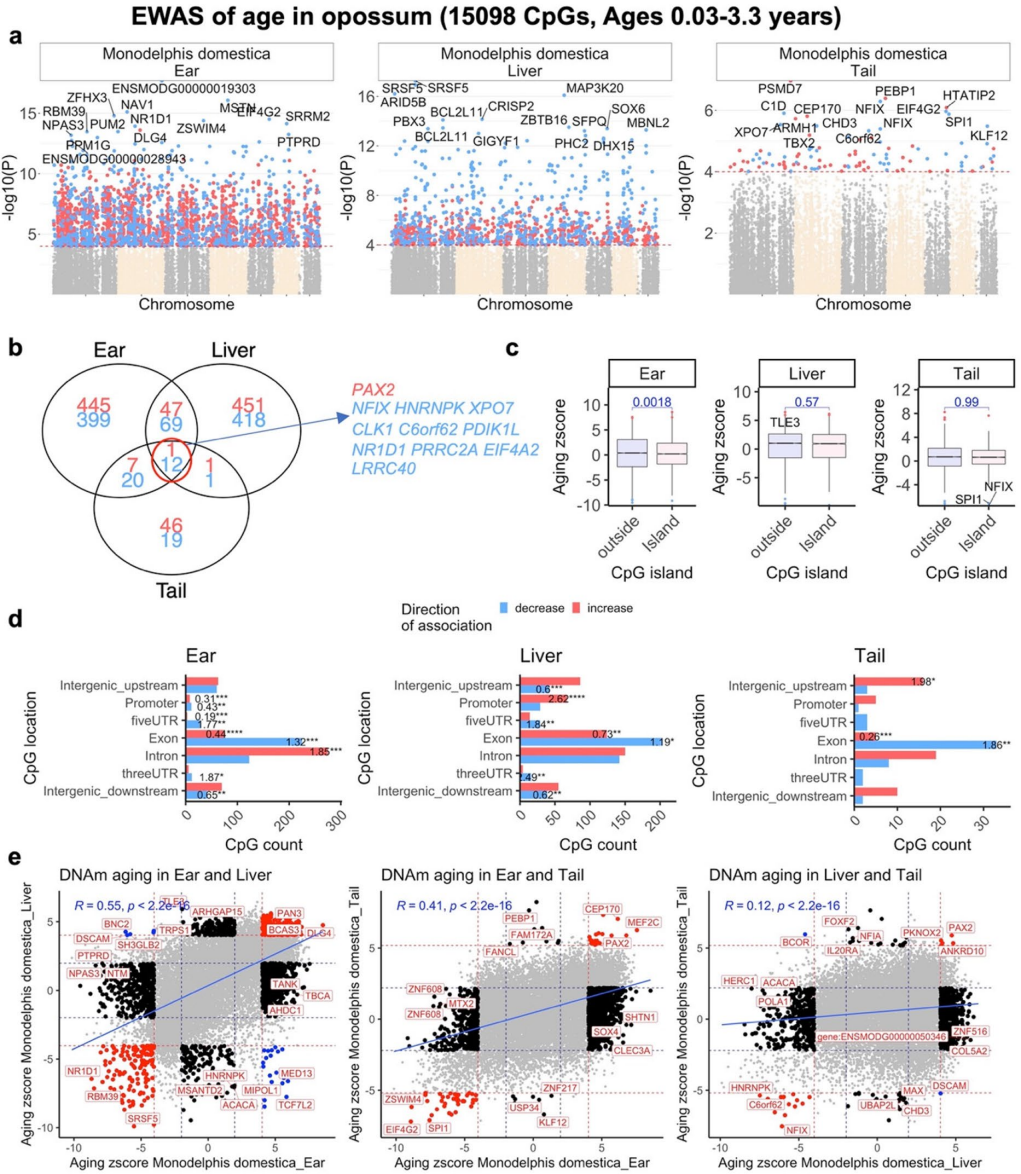
EWAS of age across the entire opossum lifespan. We analyzed all postnatal samples from opossums. EWAS of age in ear (*n* = 45), liver (*n* = 48), and tail (*n* = 7) of opossum. **a,** Manhattan plots of the EWAS of chronological age. The coordinates are estimated based on the alignment of Mammalian array probes to Monodelphis_domestica.ASM229v1.100 genome assembly. The direction of associations with *p* < 10^–4^ (red dotted line) is highlighted by red (increase) and blue (decrease) colors. Top 15 CpGs was labeled by the neighboring genes. **b,** Venn diagram of the overlap of up to 1000 significant CpGs (500 per direction) in each tissue. **c,** box plots of Z statistics from a correlation test between cytosine methylation and age (y-axis) versus CpG island status. The x axis is the Fisher transformed z score of the Pearson correlation of each CpG with age. The t-test p values are labeled above the box plots. **d,** Location of top CpGs in each tissue relative to the closest transcriptional start site. The odds ratio of the proportion changes than the background are reported in for each bar. Fisher exact *p* values: *p < 0.05, **p < 0.01, ***p < 0.001, ****p < 0.0001. **e,** Scatter plots represent the pair wise comparison of EWAS of age in different opossum tissues. The aging z scores are the Fisher z-transformation of DNAm-Age Pearson correlation for each CpG in opossum tissues. A positive (or negative) of z score means an increase (or decrease) of DNAm with age in the analyzed species. Red dotted line are the z scores corresponding to p < 10–4; blue dotted line are the z scores corresponding to *p* > 0.05; Red data dots indicate shared CpGs (i.e., The CpGs that significantly change in the same direction in both tissues) between tissue represented by the x- and y-axes; black data dots are CpG methylation changes that are significant in one but not the other tissue. The top CpGs in each sector is labeled by the identity of the adjacent genes

The mammalian methylation array contains 15,098 CpGs that map to the *Monodelphis domestica* (ASM229v1.100) genome. Due to the high inter-species conservation of the probes on the array, findings from the opossum methylation data are likely extrapolatable to humans and other mammalian species. We performed an epigenome-wide association analysis (EWAS) of age for these CpGs in the ear, liver, and tail of opossums. The EWAS analysis in tails was arguably underpowered due to low sample size (*n* = 7). At a nominal significance of *p* < 10^–4^, a total of 2971, 1328, and 107 CpGs were related to age in ear, liver, and tail tissues of opossums, respectively (Fig. 2a). The top age-associated CpGs and their proximal genes for the individual tissues are as follows: ear, a decrease of methylation in ENSMODG00000019303 promoter (p = 2 × 10^–18^) and MSTN exon (*p* = 8 × 10^–17^); liver, a decrease of methylation in SRSF5 exon (p = 5 × 10^–18^); and tail, an increase of methylation in *PSMD7* intron (p = 1 × 10^–7^). In general, age-related CpGs were enriched with genes related to RNA processing (p = 8 × 10^–15^), RNA splicing (3 × 10^–14^), cell cycle (3 × 10^–18^), and replicative senescence (1 × 10^–7^) (Fig. S4).

EWAS of chronological age revealed clear, tissue specific DNAm changes in opossums. However, these results are confounded by the different age ranges of analyzed opossum tissues. While the liver (10 days to 3.2 years) and ear (10 days to 2.2 years) were available from all ranges of opossum lifespan, the tail samples were collected only during development (10 days to 0.28 years) and the sample size was very low (*n* = 7, Table 1). Despite these caveats, our analysis identified 13 CpGs with methylation changes that are shared between these three tissues in the top 1000 age-related CpGs (500 per direction of association) (Fig. 2b).

Opossum CpG islands do not show a systematic increase of methylation with age (Fig. 2c), consistent with a unique DNAm aging profile for the species (Fig. 2c). This observation was more prominent for ear samples. In opossum ear, the CpG islands have slightly lower age correlations (measured using the Z statistic from a correlation test) than CpGs outside of islands (Student *T*-test *p* = 0.0018) (Fig. 2c). In addition, there are relatively few methylation changes in opossum CpGs located in the promoters (OD = 0.31, Fisher Exact *p* < 10^–3^) and 5’UTRs (OD = 0.19, Fisher Exact *p* < 10^–3^) of genes (Fig. 2d). These features contrast with all other mammals that have been examined to date, which generally experience age-related gain of methylation in CpG islands, promoters, and 5’UTRs [60]. Coincidentally, all previously investigated mammals happen to have been from the placental clade. To further compare the tissues, we created pairwise sector plots of DNAm aging patterns in opossum samples. We observed a high positive correlation of age-related DNAm changes between ear-liver (*r* = 0.55) and ear-tail samples (*r* = 0.45) (Fig. 2e). In contrast to these similarities, there are some age-related CpGs with methylation changes that are divergent between opossum tissues, e.g., *BNC2* (intron) shows a methylation increase in liver but a decrease in ear. This finding mirrors those from other species: aging patterns in one tissue often differ from those of other tissues.

None of the CpGs implicated in aging studies of opossums overlapped with imprinting control regions known from human studies [61].

We could not evaluate whether cytosines affect gene expression levels in opossums. However, we recently studied the relationship between cytosine methylation and gene expression levels in horses [62].

### Comparing opossum and mouse

As mentioned above, aging effects on cytosine methylation levels in the opossum differ from those of other, previously investigated mammals when it comes to their relationship with CpG island status. This observation prompted us to compare aging effects in opossum to those in mouse tissues in two age groups: postnatal development (defined as age between 0 and 6 weeks in both species) and later aging (defined as ages between 0 to > 2.5 years in both species).

#### Postnatal development in opossum and mouse

**Fig. 3:**
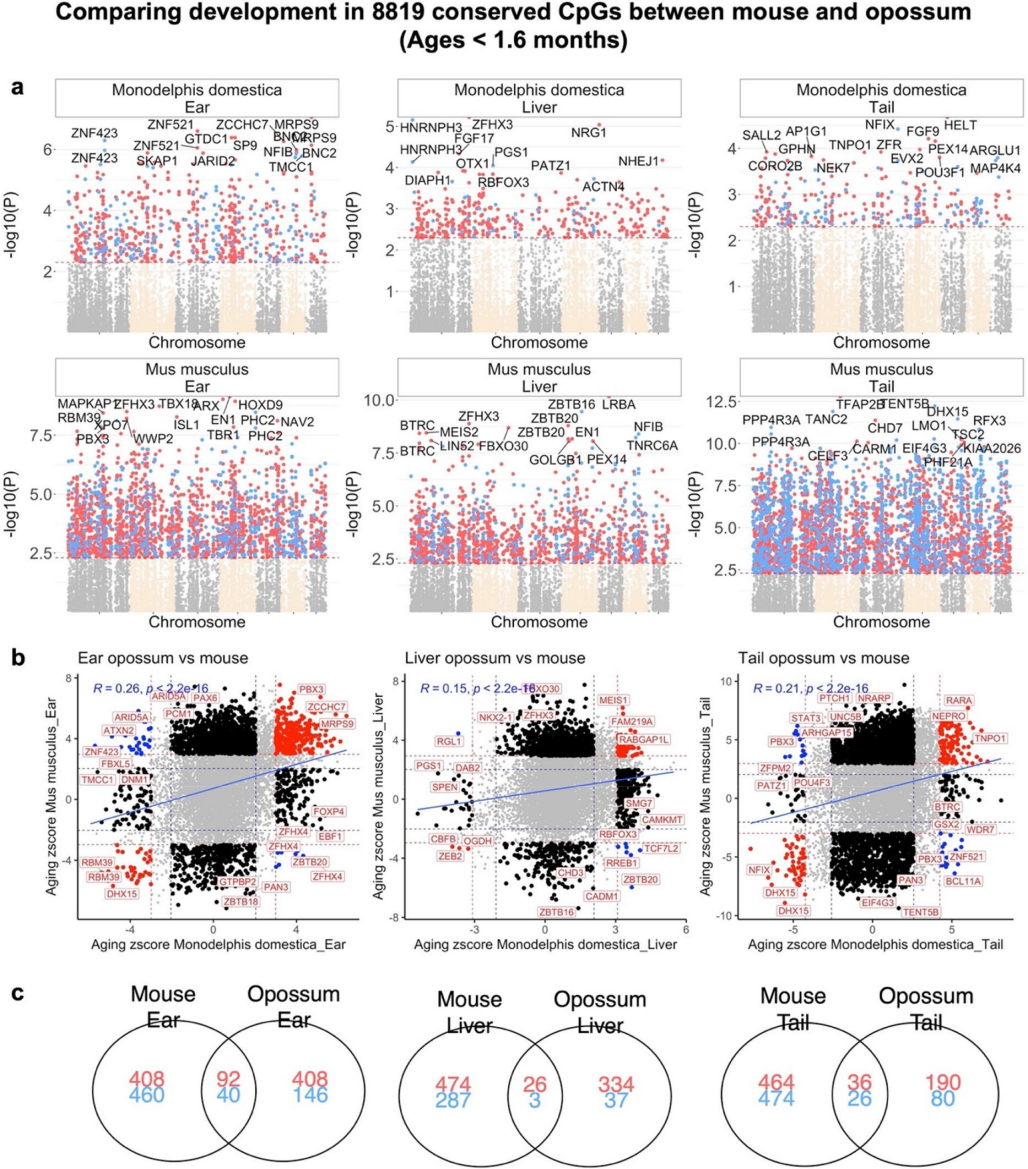
CpGs that change during development in opossums and mice. **a,** Manhattan plots of the EWAS of chronological age in developmental stages of mouse and opossum. The analysis is limited to 8819 conserved CpGs in these two species. All coordinates are reported based on Monodelphis_domestica.ASM229v1.100 genome assembly. The direction of associations with p < 0.005 (red dotted line) is highlighted by red (increased methylation) and blue (decreased methylation) colors. Top 15 CpGs was labeled by the neighboring genes based on the opossum genome. **b,** Scatter plots represent the pair wise comparison of EWAS of age in mouse and opossum. The aging z scores are the Fisher z-transformation of DNAm-Age Pearson correlation for each CpG in different tissues. A positive (or negative) z scores means an increase (or decrease) methylation with age in the analyzed species-tissue strata. Red dotted line are the z scores corresponding to *p* < 0.005; blue dotted line are the z scores corresponding to *p* > 0.05; Red dots indicates shared CpGs with methylation that change significantly in the same direction in both tissues represented by X and Y axes; black dots represent CpGs with methylation that change in one species but not the other. Gene adjacent to the top CpGs in each sector is labeled by opossum gene symbol. **c,** Venn diagram of the overlap of up to 1000 (500 in each direction) significant CpGs between mouse and opossum for each tissue. It is noteworthy that there is so little similarity between the livers of mouse and opossum, which probably reflects the substantial heterogeneity in the liver samples from opossums (Fig. S1). On the other hand, much more similarities are seen for tails of these animals, even though the number of opossum tail samples was very small (*n* = 7), which would intuitively lead one to expect lower likelihood of detecting similarities

We used a Pearson correlation test to relate CpGs to age in animals that were 6 weeks old or younger. To avoid biases due to sequence differences, we restricted the analysis to 8819 CpGs that could be mapped to orthologous genes in both species. At a significance threshold of p < 0.005 (FDR < 3 × 10^–6^ to < 0.1 depending on the species-tissue), mouse tissues (average change = 2632 CpGs) have a higher number CpGs related to age than opossum tissues (average change = 488) (Fig. 3a). This difference could have a biological origin as we have a comparable sample size, particularly in ear tissues, for these two species (16 opossum ears, 18 mouse ears). Despite this difference, there are nevertheless a considerable number of CpGs that experienced similar methylation changes in all three tissues during mouse and opossum postnatal development (mean correlation = 0.2) (Fig. 3b). In line with this, we found that 3–10% of the top 1000 age-related CpGs in mouse tissues are shared with those of opossums (Fig. 3c). These observations suggest that the epigenetic basis of postnatal development is at least partially conserved between these two, distantly related mammalian species. The shared CpGs that correlate with age during postnatal development are associated with genes related to development (*p* = 6 × 10^–10^), polycomb repressor complex 2 (PRC2) binding sites (*p* = 4 × 10^–8^), and H3K27ME3 marks (*p* = 2 × 10^–8^); a common aging pattern found in all previously analyzed mammalian species [60] (Fig. S5). The PRC2 maintains the transcriptional repressive states of genes. This complex has also been demonstrated to regulate H3K27Me3 marks, DNA damage, and the senescence of cells during aging [63]. We also identified 8 to 38 CpGs with divergent methylation patterns during postnatal development in mouse and opossum, depending on tissue (Fig. S6). These divergent CpGs are adjacent to genes involved with the immune system (e.g., lymphocyte differentiation, p = 5 × 10^–4^) and female reproductive system (e.g., luteinization, *p* = 4 × 10^–5^, Fig. S7). As expected, based on the large evolutionary distance between mouse and opossum, almost all conserved CpGs show a baseline difference between these two species at *p* < 0.005 (FDR < 0.006) significance (Fig. S8): 7360 CpGs in ear, 6510 in liver, and 7225 in tail.

#### Later aging in opossum and mouse

**Fig. 4:**
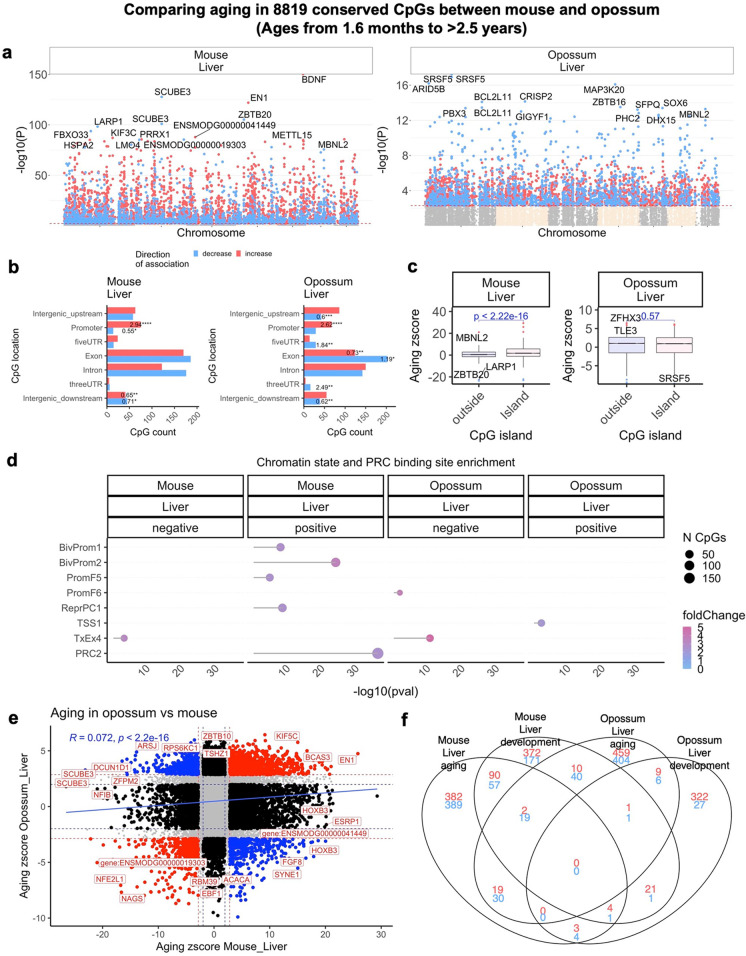
EWAS of age in adult mice and opossums. The EWAS of age analysis was limited to animals that had reached adulthood. Thus, the age at blood draw was older than the average age at sexual maturity. **a,** Manhattan plots of the EWAS of chronological age in mouse and opossum. The analysis is limited to 8819 conserved CpGs in these two species. All coordinates are reported based on Monodelphis_domestica.ASM229v1.100 genome assembly. The direction of associations with *p* < 0.005 (red dotted line) is highlighted by red (increased methylation) and blue (decreased methylation) colors. Top 15 CpGs was labeled by the neighboring genes based on the opossum genome. Sample size: mouse liver, *N* = 498, ages 0.2–2.78 years; opossum liver, *N* = 48, ages 0.03–3.3 years. **b,** Location of top age related CpGs (500 positive and 500 negative) in each species relative to the closest transcriptional start site. The odds ratio of the proportion changes than the background are reported in for each bar. Fisher exact p values: *p < 0.05, **p < 0.01, ***p < 0.001, ****p < 0.0001. **c,** box plots of DNAm aging by CpG island status. The x axis is the Fisher transformed z score of the Pearson correlation of each CpG with age. The t-test p values are labeled above the box plots. **d,** chromatin state enrichment of the top age related CpGs (500 positive and 500 negative) in each species. The enrichment *p*-values were calculated using Fisher's hypergeometric test. The chromatin states are based on the stackHMM states in human Hg19 genome. The PRC2 binding is defined by the available ENCODE ChipSeq results of EED, EZH2, and SUZ12 transcriptional factors. **e,** Scatter plots represent the pair wise comparison of EWAS of age in mouse and opossum. The aging z scores are the Fisher z-transformation of DNAm-Age Pearson correlation for each CpG in different species. A positive (or negative) z scores means an increase (or decrease) methylation with age in the analyzed species-tissue strata. Red dotted line are the z scores corresponding to *p* < 0.005; blue dotted line are the z scores corresponding to *p* > 0.05; Red dots indicates shared CpGs with methylation that change significantly in the same direction in both tissues represented by X and Y axes; black dots represent CpGs with methylation that change in one species but not the other. Gene adjacent to the top CpGs in each sector is labeled by opossum gene symbol. **f,** Venn diagram of the overlap of up to 1000 (500 in each direction) significant CpGs between liver samples of mouse and opossum during development and all age ranges

**Fig. 5:**
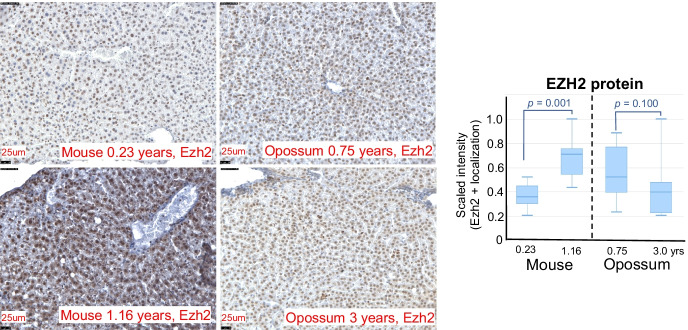
Later age-related changes in EZH2 localization in livers of mouse and opossum. EZH2 protein was studied as a marker of PRC2 activity and visualized using IHC in liver sections of young (representative section—upper left, 0.23 years of age) and old (lower left, 1.16 years) mice and young (upper right, 0.75 years) and old (lower right, 3 years) opossums. All images are shown at the same scale. As shown in the images on the left and quantified in the bar plot on the far right, Ezh2 levels significantly increase with age in mice, but not in opossum

Our datasets allowed us to further compare later aging effects in liver samples from opossum and mouse. We used *n* = 498 mouse liver samples in these comparisons. As before, we focused on 8819 CpGs that could be mapped to orthologous genes in both species. At a two-sided Student T test p value threshold of 0.005 (FDR < 0.01), a total of 6783 and 4800 CpGs are related to age in mouse and opossum respectively (Fig. 5a). The difference in the number of significant CpGs could reflect differences in sample size (*n* = 498 mouse versus *n* = 48 opossum). To avoid the potential bias arising from different numbers of significant CpGs, we limited our comparison of later aging effects to the top 1000 age related CpGs (500 per direction of association) for each species. Later aging effects on cytosines in opossum liver samples differ from those in mouse livers in several ways. Unlike what we observed in mouse, the 5 prime untranslated region and exons are significantly enriched (odds ratio = 1.84, *p* < 0.01 and OR = 1.19, *p* < 0.05, respectively) with CpGs that have a negative correlation with later aging in opossum. Furthermore, in contrast to mouse, CpGs located in opossum CpG islands do not show a higher positive association with later aging than those outside of islands (Fig. 4c).

To provide a more detailed characterization of later aging related CpGs in opossums, we sought to characterize chromatin features within which significant age related CpGs are positioned. Since chromatin states that are specific to the opossum genome are presently unavailable, we used the chromatin states (stackHMM) that identifies chromatin features based on the consensus of over 100 human cell types [55]. Despite the species difference, this approach remains informative because the design of the mammalian methylation array was based on DNA loci that are conserved across mammals [48], allowing chromatin features identified by stackHMM to be applied to mice and to a lesser extent to opossums. The chromatin state analysis reveals another unique aspect of later aging effects in opossum; while CpGs that gain methylation with later aging in mice overlap significantly with CpGs located in bivalent promoters and regions bound by polycomb repressor complex 2 binding, this is not the case for opossums (Fig. 4d). Later aging effects on cytosine levels in liver samples from mice are also only weakly correlated with those in opossums (*r* = 0.07, Fig. 4e).

To further contrast later aging in mouse with that of opossum, we analyzed EZH2 protein levels in their livers using IHC. We found that levels of Ezh2 localization are significantly higher in old mice (84 weeks/1.16 years) relative to young adult (0.23 years/12 weeks) mice (*p* = 0.001), but similar in old (3 years) and young adult (0.75 years/9 months) opossums (*p* = 0.100, Fig. 5).

In mice, we observe a significant overlap between CpGs correlated with age after sexual maturity and CpGs that correlate with age during postnatal developmental (17.3% of the top age related CpGs overlap with the top development related CpGs, Fig. 4f). However, this is not the case in opossums: only 1.6% of top age related CpGs overlap with the top development related CpGs in opossums. Developmental effects in mouse livers exhibit a weak but statistically significant positive correlation with those in opossums (Pearson *R* = 0.15, Student *T* test *p* < E-16, Fig. 3b). By contrast, later aging effects exhibit a much weaker correlation (*R* = 0.072, Fig. 4e) between the two species.

### Later aging effects in other marsupial species

Our EWAS of later aging in opossum led to unexpected results, e.g., CpGs located in CpG islands are not different from those outside of islands when it comes to age related gain of methylation. To evaluate whether this unexpected result is also present in other marsupial species, we analyzed data from five marsupial species from the taxonomic orders Dasyuromorphia and Diprotodontia, which diverged 81 million years ago from the order Didelphimorphia, which contains opossum. Thus, our analysis covers phylogenetically distant marsupial species and is not biased toward a single branch of the infraclass Marsupialia. Our access to tissues samples for Diprotodontia and Dasyuromorphia was limited to blood (Methods). Because of this, and because we did not have access to blood samples for opossums, we also performed a pair-wise comparison of later aging effects in these marsupial species to mouse blood.

**Fig. 6:**
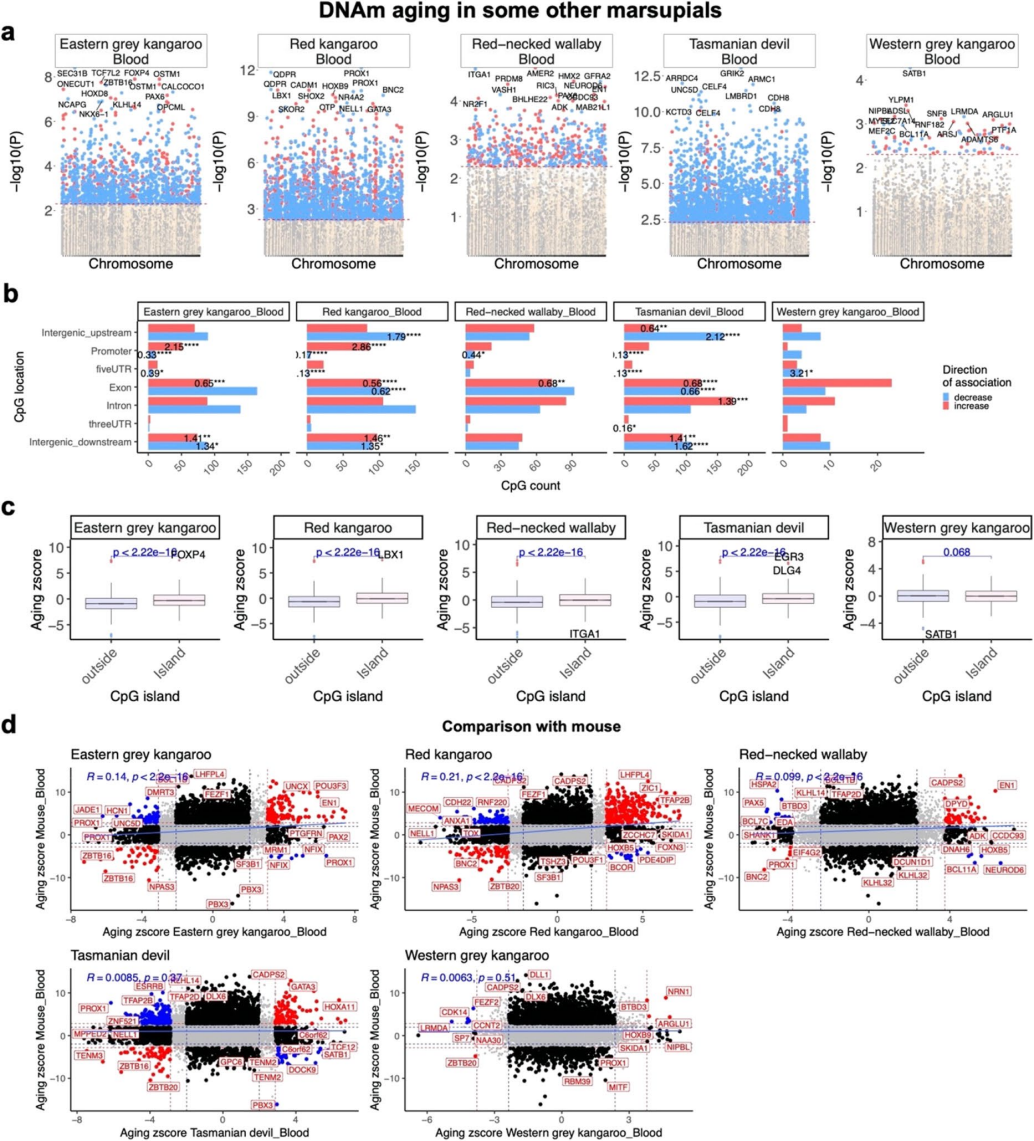
Epigenetic aging effects in other marsupial species. **a,** Manhattan plots of the EWAS of chronological age in the blood of five marsupial species. All coordinates are reported based on *Phascolarctos cinereus* (phaCin v4.1.100) genome assembly. The direction of associations with *p* < 0.005 (red dotted line) is highlighted by red (increased methylation) and blue (decreased methylation) colors. Top 15 CpGs was labeled by the neighboring genes based on the opossum genome. Sample size: Eastern grey kangaroo, *N* = 12, ages: 1.8–13.3 years; Red kangaroo, *N* = 37, age: 0.6–11.7 years; Red-necked wallaby, *N* = 5, ages: 2–9.6 years; Tasmanian devil, *N* = 41, ages 0.5–8 years; Western grey kangaroo, *N* = 5, ages: 1.6–5 years. **b,** Location of top age-related CpGs (500 positive and 500 negative) in each species relative to the closest transcriptional start site. The odds ratio of the proportion changes than the background are reported for each bar. Fisher exact *p* values: *p < 0.05, **p < 0.01, ***p < 0.001, ****p < 0.0001. **c,** box plots of DNAm aging by CpG island status. The x axis is the Fisher transformed z score of the Pearson correlation of each CpG with age. The t-test p values are labeled above the box plots. **e,** Scatter plots represent the pair wise comparison of EWAS of age in mouse and other marsupial species. This analysis is limited to 10,968 CpGs conserved between mouse and *Phascolarctos cinereus* genomes. The aging z scores are the Fisher z-transformation of DNAm-Age Pearson correlation for each CpG in different species. A positive (or negative) z scores means an increase (or decrease) methylation with age in the analyzed species-tissue strata. Red dotted line are the z scores corresponding to *p* < 0.005; blue dotted line are the z scores corresponding to *p* > 0.05; Red dots indicates shared CpGs with methylation that change significantly in the same direction in both tissues represented by X and Y axes; black dots represent CpGs with methylation that change in one species but not the other. Gene adjacent to the top CpGs in each sector is labeled by opossum gene symbol. Mouse blood *N* = 97, ages: 0.1–2.25 years

For EWAS of later aging in dasyurid and diprotodontian marsupials, we used alignments based on the *Phascolarctos cinereus* (koala) genome rather than that of opossum, because koala is evolutionarily closer to these species. The mammalian methylation array contains 17,856 CpGs that can be mapped to koala genome. At a nominal significance of *p* < 0.005 (FDR < 0.01 to < 1e-9 depending on the sample size), a total of 91 to 2356 CpGs are associated with later aging, based on species and sample size (Fig. 6a, Table S10). Strikingly, the pattern of later aging effects in these marsupials are more similar to those of placental mammals than to those of opossums. For example, none of these additional marsupials exhibit an age-related decrease of methylation in CpGs located in 5`UTR and exons (Fig. 6b). In addition, CpG islands show a systematic gain of methylation with later aging relative to non-island CpGs in these marsupials, similar to the pattern found across placentals but not in opossum (Fig. 6b). The enrichment of the genes with PRC2 complex binding sites was also observed in these marsupials but not opossum (Fig. S10). Two of the kangaroos (Eastern grey and red kangaroos) also show a high, positive DNAm aging correlation (*R* = 0.14 to 0.21) with mouse that is higher than that observed in opossum (Fig. 6c).

## Discussion

While the Mammalian Methylation Consortium has constructed pan mammalian clocks that can be applied across all mammalian species [60], species-specific clocks are generally expected to be more accurate. We previously developed dual species clocks for several species of placental mammals [32–38]. In this study, our employment of the mammalian array to profile three tissues from 100 opossums allowed us to construct a pan-tissue opossum clock, our first clock for a member of the marsupial clade. This clock should be very useful in research that employs opossum as the animal model of choice. Similarly, we developed epigenetic clocks for additional marsupials: the Tasmanian devil and the genus *Macropus*, i.e., kangaroos and wallabies (Fig. 1).

The translational utility of an opossum clock could be further enhanced if it also applied to DNA from humans. This would ease the translation of findings from the opossum to human and vice versa. This rationale, which previously drove us to create dual species clocks in numerous other animals, led us to develop human-opossum clocks. To achieve this goal, in addition to DNA sequence differences between humans and opossums, we had to also contend with markedly different lifespans between these species. The maximum lifespan of opossum (*Monodelphis domestica*) is only 4.2 years according to the “anAge” data base [50, 51], which is 29 times shorter than that of humans (maximum lifespan 122.5). This is an important consideration because the biological fitness of a 3-year-old opossum is not equivalent to that of a 3-year-old human. We addressed this challenge by incorporating the concept of relative age. An opossum with a relative epigenetic age of 0.5 is much more comparable to a human of the same relative age. Following this logic, we generated a human-opossum clock for relative age that can be applied to both humans and opossum-based models of diseases and health conditions. These clocks will facilitate investigations into the epigenetic correlates of development and aging and allow evaluation of the impact of environment, living condition, food, and treatment on the rate of opossum epigenetic aging, which will be translatable to human aging.

While the CpGs that constitute the clocks are the most accurate with regards to age estimation, they are not the only CpGs that display methylation changes that alter with age. Indeed, the clock CpGs comprise only a small subset of all age-related CpGs. Hence, efforts to accrue biological meaning of age-related methylation changes necessitate that all age-related CpGs are analyzed in epigenome-wide association studies (EWAS). Such analyses in opossum uncovered some unexpected features; one of the most notable being the absence of increased methylation of CpG islands with later aging (Fig. 2c). We found this finding to be particularly interesting as all mammalian species that had been studied up to the time of our analyses exhibit this feature, and all previously studied species happened to have been from the placental clade. This suggested that marsupials and placentals might differ in this trait, and in the processes through which they biologically age as adults. However, our further studies documented the expected age-related gain of CpG island methylation in other marsupials (e.g., kangaroos, wallabies, Tasmanian devils) (Fig. 5c), suggesting that the opossum pattern is not broadly shared across marsupials. Taken together, these findings are consistent with the absence of increased methylation of CpG islands with later aging in opossum being a unique, possibly species- to order- level trait, or a false positive result.

If the methylation of CpG islands was the only way in which the opossum profile stood apart from that shared by other mammals, we would be more inclined to consider this result a false positive. However, opossums also stand apart in their lack of a later aging-related gain of methylation in PRC2 binding sites. All examined placentals and other marsupials (e.g., kangaroos, wallabies) show such a gain during later aging [60]. Our Ezh2 IHC results provide additional support for the hypothesis that PRC2 activity increases with later aging in mouse tissues, and potentially also in the tissues of other mammals, but remains constant in the opossum tissues. Opossum also shows a loss of methylation in exons with later aging, another pattern not observed in mouse liver (Fig. 4c) or in the other marsupials (Fig. 5b) in this study. We also only observe a weak consistency in later aging patterns between opossums and mice when looking at individual cytosines (Fig. 4e).

Opossum’s unique CpG island and PRC2 methylation profiles during later aging might be a result of the unique nature of the opossum genome and might be linked. The opossum genome, like that of many other marsupials, is of comparable size to those of placentals, but packaged into fewer, larger chromosomes (opossum 2n = 18) [5]. Macropodids and Tasmanian devils, the other marsupials included in this study, have 2n = 22 and 2n = 14 chromosomes, respectively, while mouse has 2n = 40 and human 2n = 46. However, seemingly unlike other examined marsupials, opossum also possesses a relatively low level of CpG content, on the order of half or less the content of many other amniotes, and a correspondingly low density of CpG islands (7.5 per Mb) [5, 64, 65]. CpG content in tammar wallaby, for example, is similar to that in humans [66]. Opossum’s low density of CpG islands could also be impacting PRC2 activity, as detectable PRC2 binding primarily overlaps with CpG islands in those mammals in which it has been studied, e.g., mouse and humans [67, 68]. However, a unique genomic configuration cannot explain the association of PRC2 binding with postnatal development but not later aging in opossum. Therefore, while results of this study are intriguing, more research is needed to resolve these questions.

Our analyses revealed a high degree of tissue-specific, age-related methylation changes in later aging opossums. This is to be expected as methylation is a means by which expression of genes are regulated in different tissues. At this early stage of our understanding of epigenetics, it is not possible to determine from first principles which CpG methylation events impact gene expression and, if they do, of which genes. Hence, we employed a conservative approach of linking CpGs to the potential regulation of genes that are adjacent and proximal to them. This identified distinct sets of potential age-related genes in the ear, liver, and tail. One of the highest scoring potential age-related genes of the ear is *MSTN*, which encodes myostatin. This is of note because myostatin level rises with age in humans, with the highest levels in frail individuals [69]. This is connected to myostatin’s association with muscle loss and dysfunction of muscle stem cells [70]. In the tail, the highest scoring age-related CpG is adjacent to the gene *PSMD7*, which is a subunit of the proteasome and essential for the destruction of incorrectly folded and dysfunctional proteins. This is of potential interest as reduced efficiency of protein homeostasis is a major hallmark of aging. Furthermore, reduced expression of *PSMD7* leads to reduced mTOR level and activity, and perturbation of mTOR activity has been demonstrated to impact the rate of epigenetic aging [71]. It is important to note that at this stage of understanding, these connections uncover only potential associations. They do not yet allow us to know the direction of gene expression changes and the outcomes. The complexity of these changes can be further compounded by candidate genes that encode proteins with potentially higher levels of impact. As a case in point, in the opossum liver, the highest scoring age-related CpG is proximal to *CRCF5*, which encodes a splicing factor that carries out alternative splicing of precursor mRNAs. Such a candidate protein could induce cellular impacts that are orders of magnitude greater since they affect the expression of many other genes. Elucidation of potential age-related outcomes of such epigenetic changes is very complex and requires empirical investigation.

Another set of potential age-related proteins with equally complex outcomes are transcription factors. These proteins also affect the expression many other genes, making a non-empirical deduction of cause to effect impossible. As a case in point, one of the highest-scoring age-related CpGs common to all three opossum tissues during later aging lies next to the *PAX2* gene. *PAX2* encodes a transcription factor that is a member of PAX protein family, and proteins of this family play many critical roles in the regulation of developmental genes across species. Indeed, *PAX2* was also identified as an age-related gene in an EWAS carried out in baboons and other members of the Pax family have been implicated in the epigenetic aging of other species [43]. Two other high-scoring, age-related CpGs shared between all three opossum tissues during later aging lie adjacent to the *NFIX* gene, which encodes another transcription factor. Mutation of the *NFIX* gene in humans is associated with Sotos Syndrome 2; a developmental disorder characterized by excessive growth and advanced bone age, and patients exhibit accelerated epigenetic aging [72]. These results highlight the strong epigenetic association of the processes of development and later aging, an association that has been broadly recovered across members of the mammalian clade [60]. Collectively, the conservative approach of looking at genes that are proximal to age-related CpGs have uncovered multiple, tantalizing potential age-related genes in adult opossums. It is acknowledged that epigenetic changes can have distal effects too, and when the technology and methodology to analyze these become available, we will be able to generate a more comprehensive understanding of epigenetic aging.

In contrast to the dramatic differences we observed in the methylation profiles of adult opossums and mice during later aging, the profiles of these species during postnatal development are much more similar. During postnatal development, age-related CpGs in both mouse and opossum are located near genes associated with developmental processes and PRC2 binding sites, among other processes. These findings suggest that the processes shaping postnatal development are at least partially conserved in these species, and given these species’ deep evolutionary divergence, perhaps also among most mammals. The patterns observed in opossum and mouse during postnatal development are also reminiscent of those observed in mouse and other species during later aging, but not in opossum. This further supports the existence of a general connection between the epigenetic processes governing development and aging across most mammals. Comparisons of postnatal development in these species also discovered some potentially biologically relevant differences, including a strong association of age and CpGs located near immune system-related genes in opossums but not mouse. In contrast to placentals, much of the development of the adaptive immune system occurs after birth in marsupials [73, 74]; for example, opossum newborns do not begin to produce their own antibodies until a week or more after their birth [75]. As a result, newborn marsupials must navigate the ex-utero world and its potentially pathogenic microorganisms with only their innate immune system active [76]. Survival of the newborn in this relatively unprotected state is thought to depend heavily on antimicrobial peptides obtained through the mother’s milk [77–80]. Consistent with this, the marsupial genome displays expansions in some components of the innate immune system, including the cathelicidin and defensin families of antimicrobial peptides [75, 77].

In conclusion, we present here new epigenetic clocks for opossum that will be of use and interest to those who employ these unique creatures as animal models. We also found evidence that the methylome of opossums is distinct from that of all other examined mammals during later aging, but more similar during postnatal development. The potentially age-related genes we identified provide a foundation for further investigation of the biology and aging of these marsupials, and the human-opossum clocks we generated will be instrumental in translating any future findings to human aging. Finally, we generated the first epigenetic clocks for Tasmanian devil, red kangaroos, and animals from the genus *Macropus*; thereby adding another tool to the conservation toolbox for the management of these species.

## Supplementary Information

Below is the link to the electronic supplementary material.

Supplementary file1 (XLSX 1700 KB)

Supplementary file2 (DOCX 3009 KB)

## Data Availability

New data for epigenetic clock studies can be generated with the mammalian methylation array, which is distributed by the Epigenetic Clock Development Foundation: https://clockfoundation.org/ The data will be made publicly available as part of the data release from the Mammalian Methylation Consortium. Genome annotations of these CpGs can be found on Github https://github.com/shorvath/MammalianMethylationConsortium
