# Supplementary material for: Epigenetic clock and methylation studies in marsupials: opossums, Tasmanian devils, kangaroos, and wallabies
Source: GeroScience. 2022 Apr 21;44(3):1825–45. doi: 10.1007/s11357-022-00569-5 (PMC9213610; doi:10.1007/s11357-022-00569-5)
Supplement: Supplementary file 2 — Supplementary file2 (DOCX 3009 KB) [file 11357_2022_569_MOESM2_ESM.docx]

**SUPPLEMENTARY MATERIAL**

for ***"Epigenetic clock and methylation studies in marsupials: opossums, kangaroos, Tasmanian devils, and wallabies".***

**Figure S1.** **Unsupervised hierarchical clustering in opossum**. Average linkage hierarchical clustering based on the interarray correlation coefficient (Pearson correlation). The first color-band underneath the tree color codes tissue: ear (turquoise), liver (blue), tail (brown). Age color codes numeric values: old (red) versus young (white). The third color band encodes sex (female= pink, male=lightblue). The opossum tissues largely cluster by tissue type but the liver samples (blue) fall into 3 separate branches (clusters) at a height cut-off (y-axis) of say 0.05. The fact that the liver samples don't cluster together could reflect technical noise (resulting from thawing the entire frozen animal) or biological variability.

**Figure S2.** **Unsupervised hierarchical clustering in mice**. Average linkage hierarchical clustering based on the interarray correlation coefficient (Pearson correlation). The first color-band underneath the tree color codes tissue: Blood (turquoise), ear (blue), liver (brown), muscle (yellow), tail (green), whole brain (red). Age color codes old (red) versus young (white). Female= pink, male=lightblue.


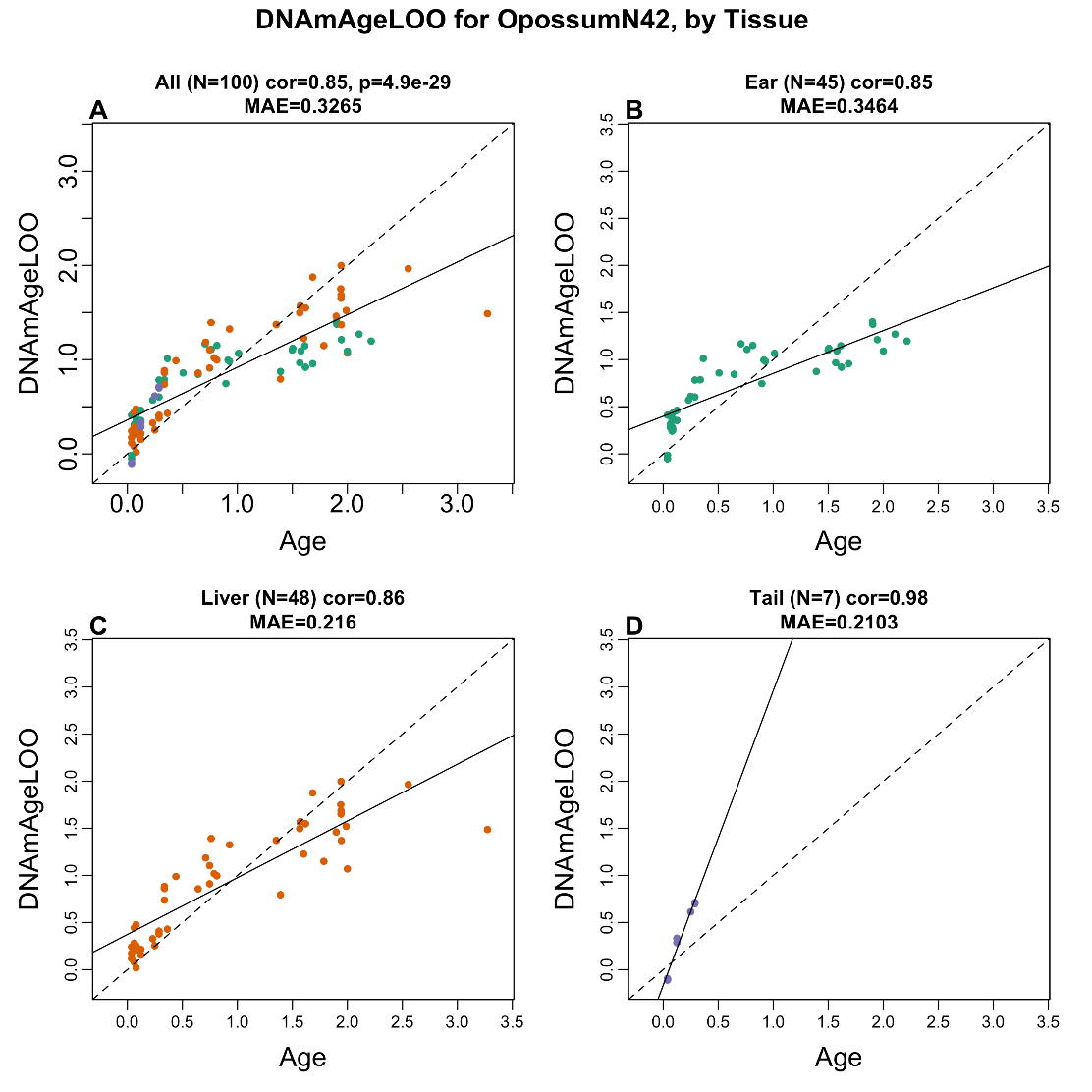


**Figure S3. Pan tissue clock for opossums**. Leave one sample out (LOO) estimates of age (y-axis) based on methylation levels versus chronological age at sample collection (x-axis). All axes are in units of years. A) All tissues combined. Dots (samples) are colored as in the other panels. B) ear samples (green dots),C) liver samples (red dots) ,D) tail samples (blue dots). The title of each panel reports the tissue, the sample size (N), the Pearson correlation coefficient, and the median absolute error.


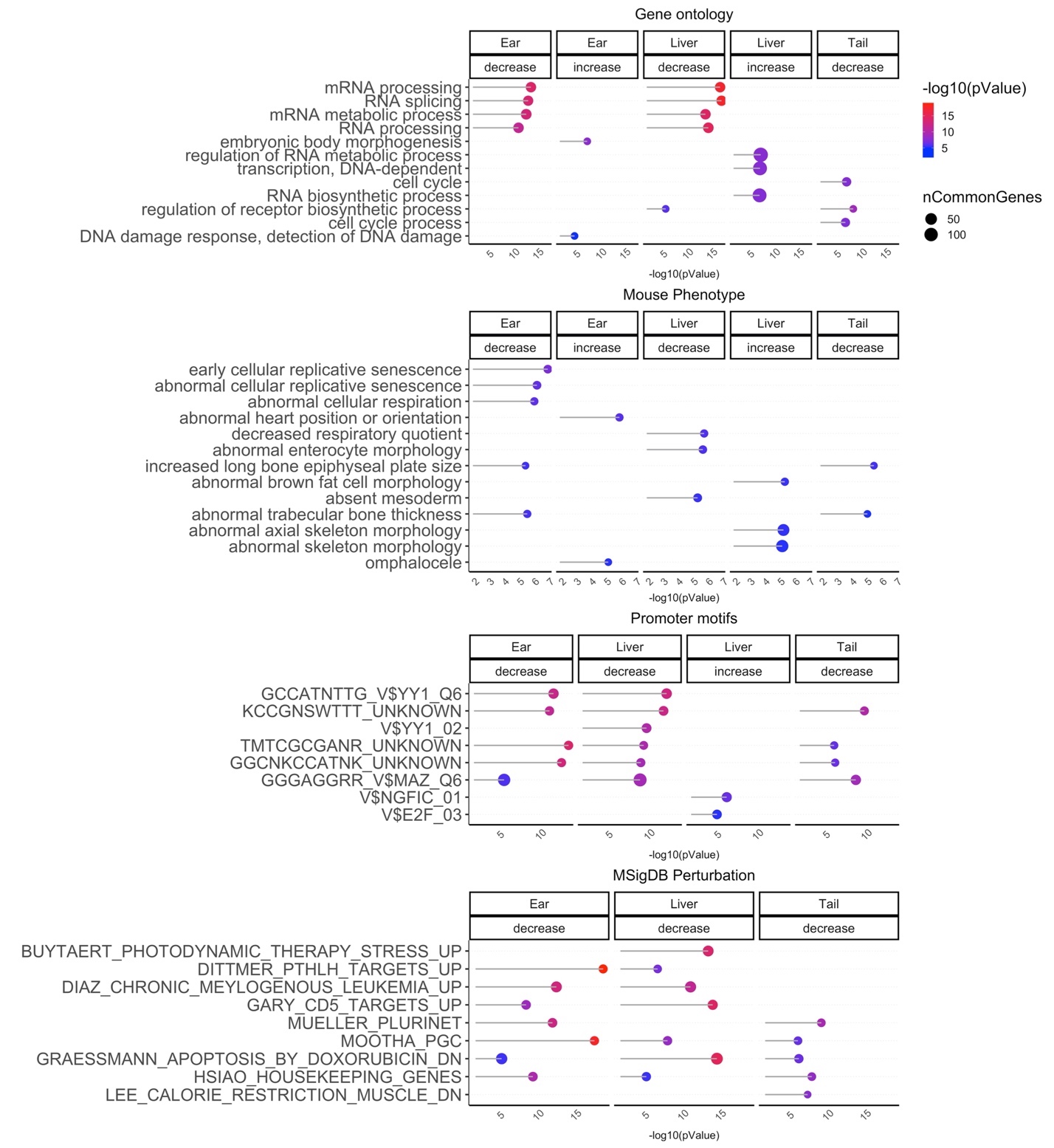


**Figure S4. Gene set enrichment analysis of age related CpGs in opossum tissues** . The gene level enrichment was done using GREAT analysis [1] and to CpGs that could be aligned to opossum. The CpGs were annotate with adjacent genes in 50kb flanking region. We extracted up to top 500 CpGs based on p value of association per direction of change as input for the enrichment analysis. The p values are calculated by hypergeometric test of the EWAS results with the genes in each background dataset. Datasets: gene ontology, mouse phenotypes, promoter motifs, and MsigDB Perturbation, which includes the expression signatures of genetic perturbations curated in GSEA database. The results were filtered for significance at p < 10^-3^ and only the top terms for each EWAS result.


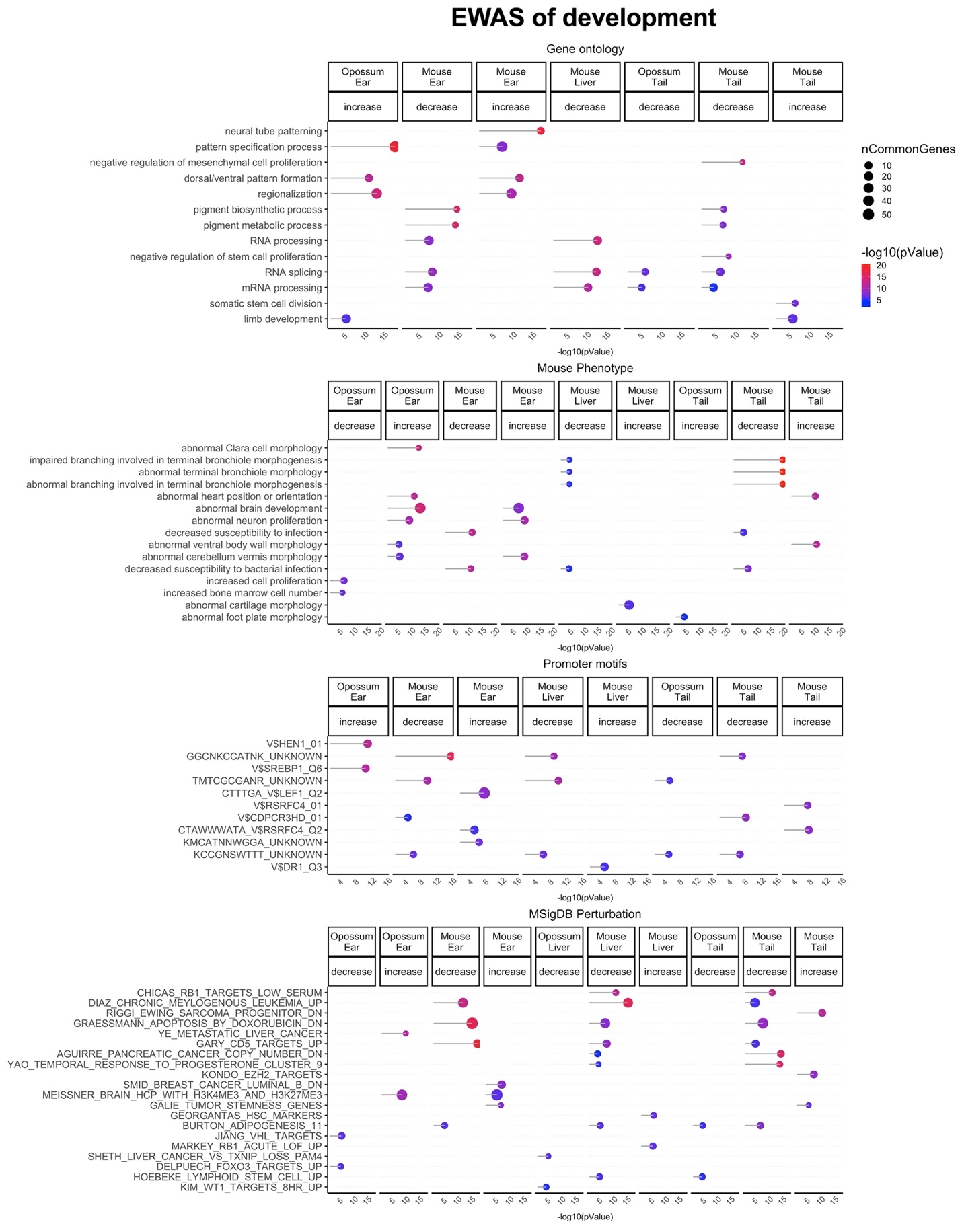


**Figure S5.** **Gene set enrichment analysis of age related cytosines in opossum tissues**. The gene level enrichment was done using GREAT analysis [1] and limited to 8819 conserved CpGs between mouse and opossum. The CpGs were annotated by adjacent genes in the 50kb flanking region. As input, we used up to top 500 CpGs based on p-value per direction (age related gain/loss of methylation). The enrichment p-values were calculated using the GREAT analysis. Datasets: gene ontology, mouse phenotypes, promoter motifs, and MsigDB Perturbation, which includes the expression signatures of genetic perturbations curated in GSEA database. Points are colored by significance level as indicated in the legends. All terms are significant at a nominal p value < 10^-3^.


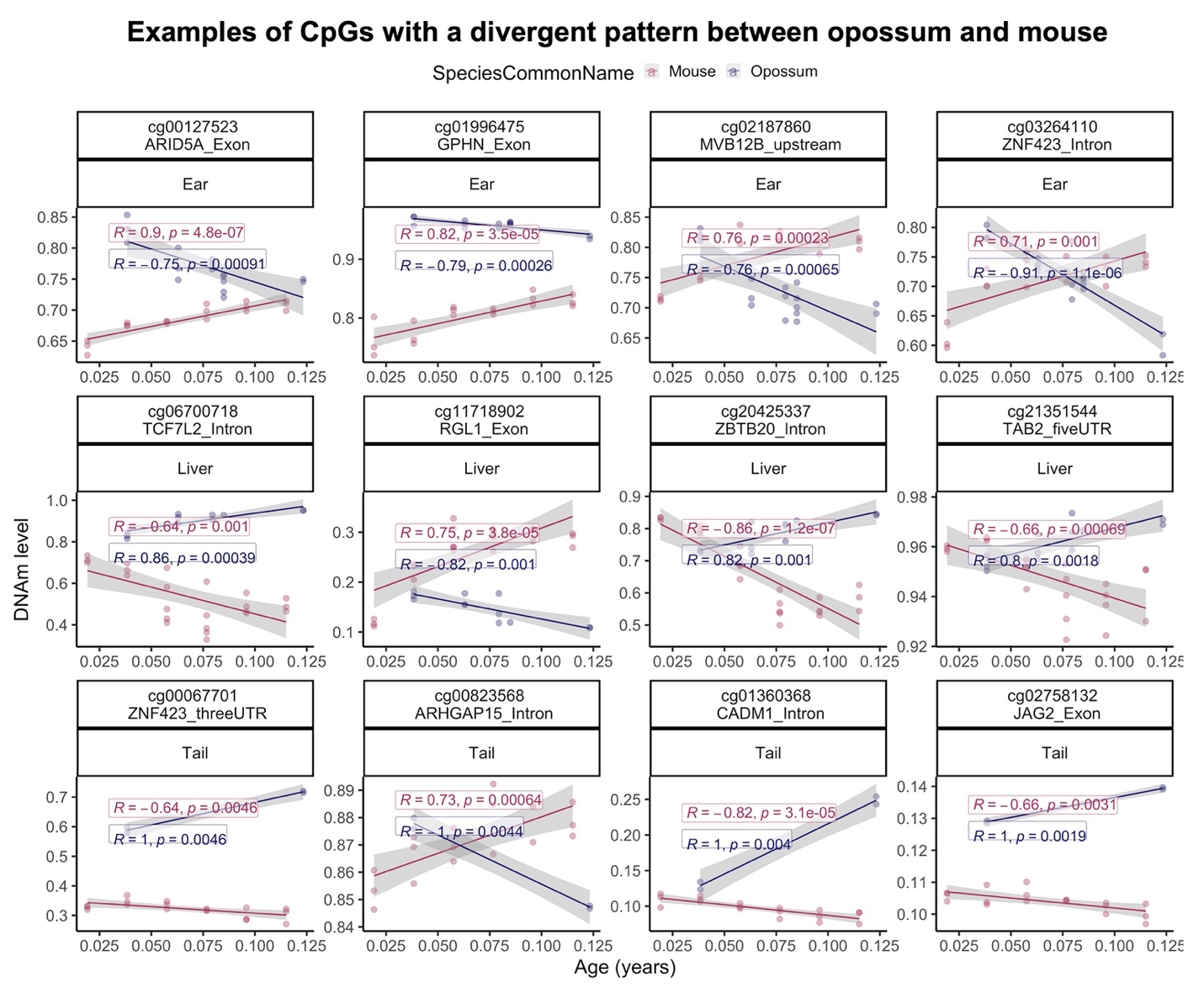


**Figure S6.** **CpGs whose developmental patterns differ between opossums and mice.**

Each panel corresponds to a different CpG whose developmental pattern (aging pattern during development) differs between the two species. The methylation values (beta values, y-axis) versus chronological age (in years, x-axis). Each dot corresponds to a DNA sample colored by species (red=mouse, blue=opossum). The lines correspond to linear regression lines. Each panel reports the tissue type, Pearson correlation coefficients and Student T test p values.


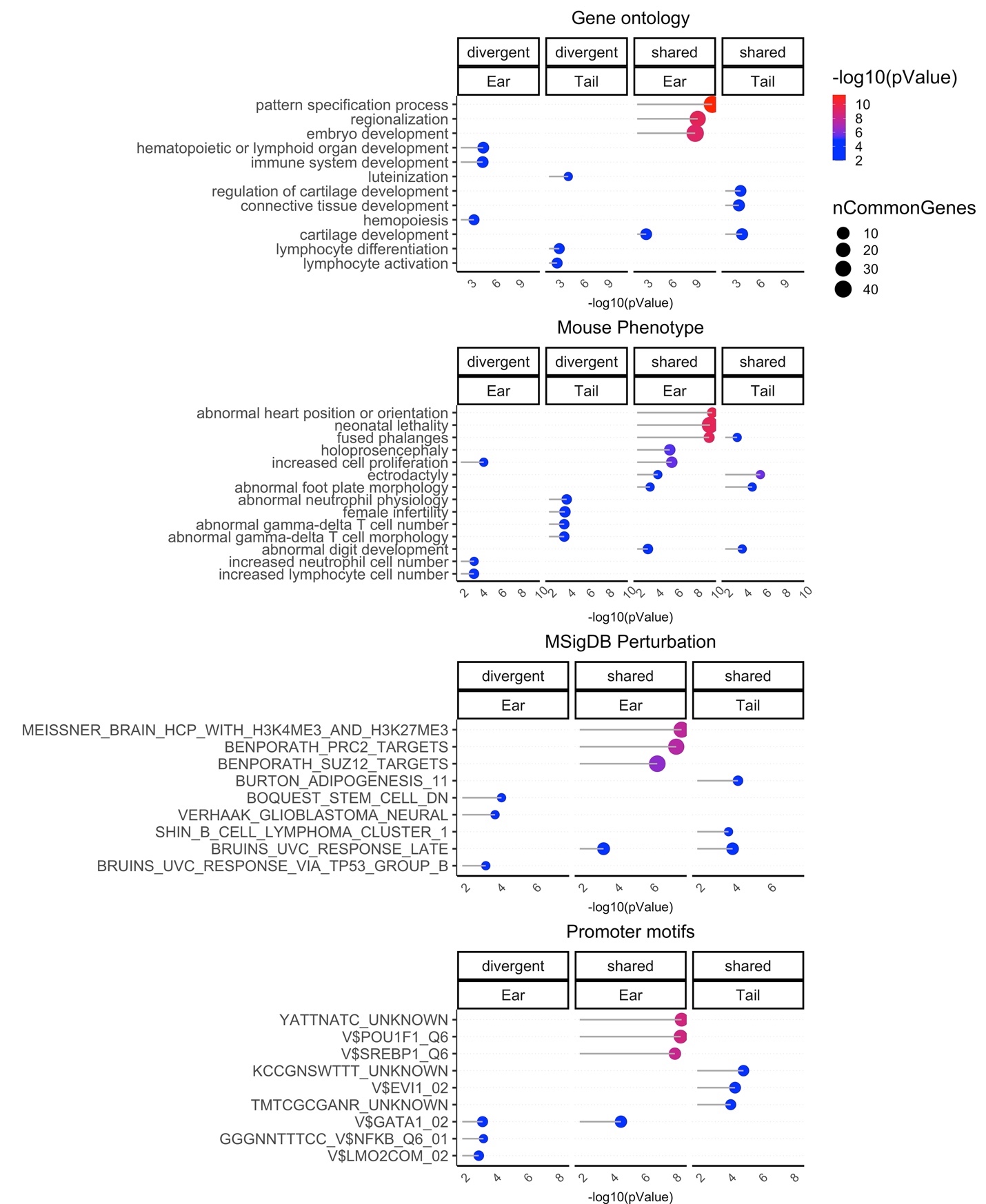


**Figure S7. Gene set enrichment analysis of cytosines that relate to development in mouse and opossum**. The gene level enrichment was done using GREAT analysis [1] and limited to 8819 conserved CpGs between mouse and opossum. The CpGs are annotated with adjacent genes within 50kb. We extracted up to top 500 CpGs based on p value of association per direction of change as input for the enrichment analysis. The p values are calculated by hypergeometric test of the EWAS results with the genes in each background dataset. Datasets: gene ontology, mouse phenotypes, promoter motifs, and MsigDB Perturbation, which includes the expression signatures of genetic perturbations curated in GSEA database. The results were filtered for significance at p < 10^-3^ and only the top terms for each EWAS are reported. The shared set means a similar age-related change in both species. The divergent group indicates the CpGs with an opposite age-related change between mouse and opossum.


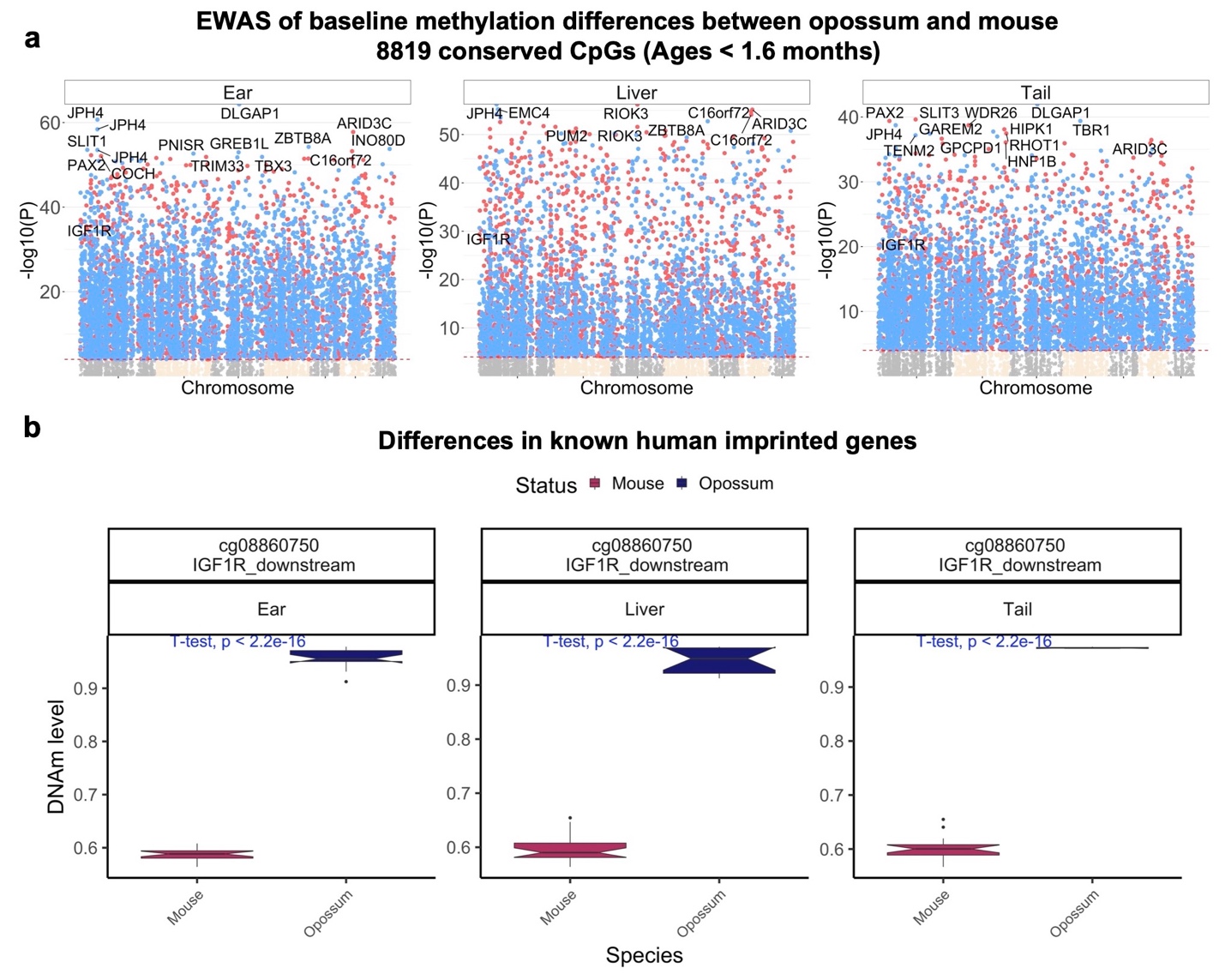


**Figure S8. Baseline (mean) methylation differences between mouse and opossum tissues. a,** Manhattan plots of the EWAS of species in developmental stages of mouse and opossum. The analysis is limited to 8819 conserved CpGs in these two species. All coordinates are reported based on Monodelphis_domestica.ASM229v1.100 genome assembly. The direction of associations with p<0.005 (red dotted line) is highlighted by red (increased methylation) and blue (decreased methylation) colors. Top 15 CpGs was labeled by the neighboring genes based on the opossum genome. In addition, IGF1R, as a known imprinted gene, is also highlighted in the figure. The species EWAS was done by a multivariate linear model including age as a co-variate. **b,** Box plot of the methylation difference between mouse and opossum in a CpG adjacent to IGF1R gene.


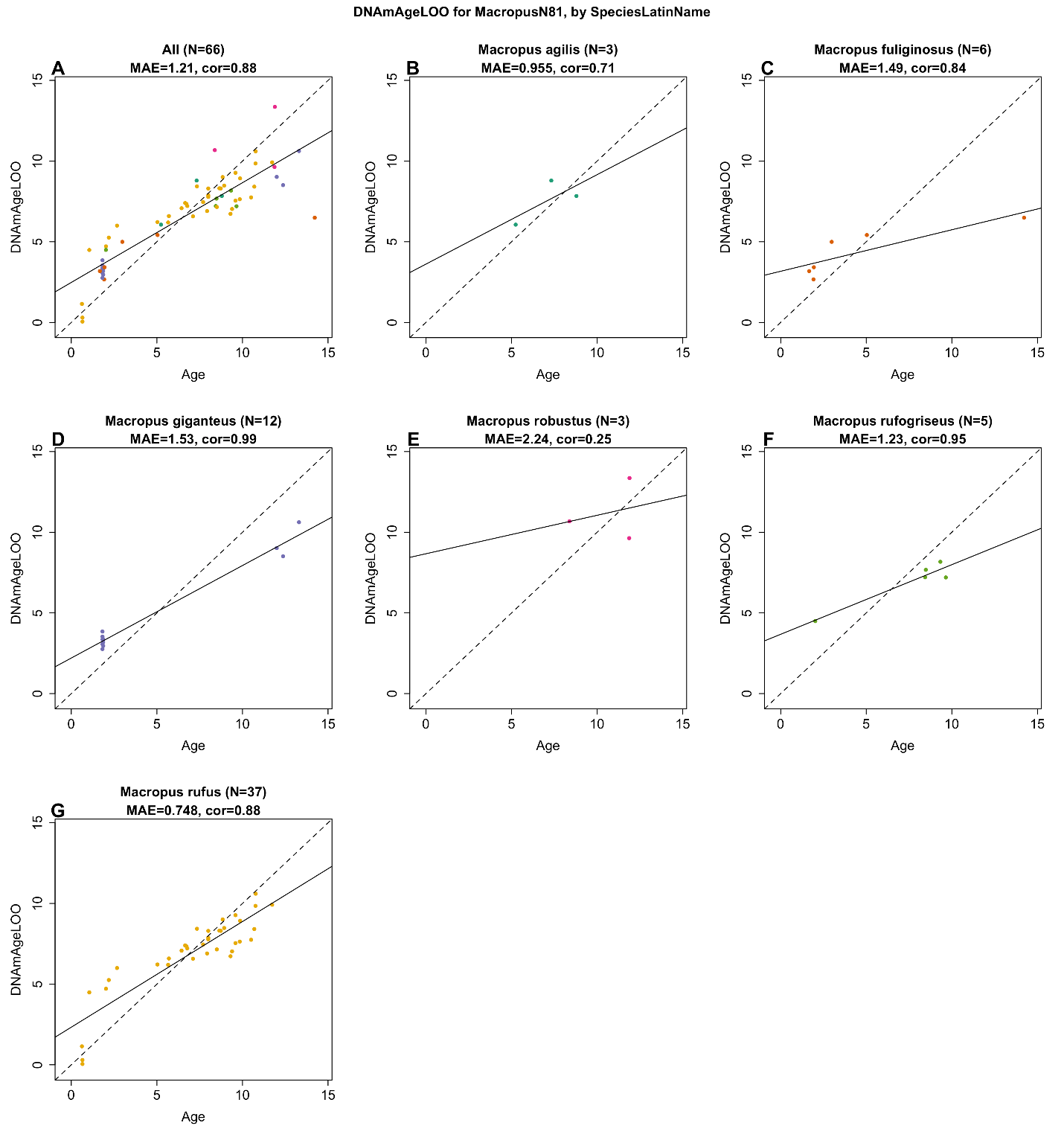


**Figure S9. Epigenetic clock for Macropus applied to specific species.**

Leave one out analysis cross validation estimates of DNA methylation age in blood samples from the genus Macropus. A) All blood samples combined. B-G) Subset of CpGs corresponding to specific species. Blood clock for the genus Macropus. Due to the low sample size, we combined all blood samples from species in the marsupial genus Macropus (i.e. species in the family Macropodidae). Specifically we used blood samples from Macropus rufus (Red kangaroo), Macropus giganteus (Eastern grey kangaroo), Macropus fuliginosus (Western grey kangaroo), Macropus rufogriseus (Red-necked wallaby) as detailed in Table 1. Each panel reports the median absolute error, Pearson correlation, and sample size (N).

**Figure S10.** **Gene set enrichment analysis of DNAm aging in marsupial tissues**. The gene level enrichment was done using GREAT analysis [1] and limited to 8819 conserved CpGs between mouse and opossum. The CpGs were annotated by adjacent genes in the 50kb flanking region. As input, we used up to top 500 CpGs based on p-value per direction (age related gain/loss of methylation). The enrichment p-values were calculated using the GREAT analysis. Datasets: gene ontology, mouse phenotypes, promoter motifs, and MsigDB Perturbation, which includes the expression signatures of genetic perturbations curated in GSEA database. Points are colored by significance level as indicated in the legends. All terms are significant at a nominal p value < 10^-3^.

**Technical Details surrounding the DNAm age estimator**

The epigenetic clock software for opossums (and other species) can be applied to data generated on the mammalian array platform [2]. New data for epigenetic clock studies can be generated with the mammalian methylation array (HorvathMammalMethylChip40), which is distributed by the Epigenetic Clock Development Foundation: https://clockfoundation.org/

The coefficient values and CpGs underlying the clocks can be found in **Table S7**.

Age transformations may be carried out as specified in the following.

**Statistical methods used for building the clocks**

The epigenetic clocks were used by employing a single elastic net regression model analysis (R function glmnet). We use used Leave-one-out analysis (LOO) using a single lambda value. We chose the following parameters for the glmnet R function (Alpha: 0.5, CV Fold: 10, Lambda choice for Clock: 1 standard error above minimum CV-MSE).

**Covariates and coefficient values of the Opossum clocks**

1. The Opossum pan tissue clock is based on 28 CpGs whose coefficient values are specified in the column "Coef.OpossumPanTissue" in Table S7. Age transformation=identity, i.e. F(Age)=Age
2. The human Opossum clock for chronological age is based on 506 CpGs whose coefficient values are specified in the column "Coef.HumanOpossumLogLinearAge". Age transformation=log-linear described below.
3. The final human Opossum clock for relative age is based on 498 CpGs whose coefficient values are specified in the column "Coef.HumanOpossumRelativeAge". Age transformation: relative age. i.e. F(Age)=Age/maxLifespan. Max lifespan for Opossums is 5.1 years. Human max lifespan =122.5 years.
4. Clock for ear samples from Tasmanian devil uses 28 CpGs, see Coef.TasmanianDevilEar
5. Clock for blood from species of the genus Macropus uses 42 CpGs, see Coef.MacropusBlood
6. Clock for blood from red kangaroos uses 32 CpGs, see Coef.RedKangarooBlood

**General description of age transformation**

The human-Opossum clocks for chronological age used log linear transformations that are similar to those employed for the HUMAN pan tissue (Horvath 2013) [3].

An elastic net regression model (implemented in the glmnet R function) was used to regress a transformed version of age on the beta values in the training data. The glmnet function requires the user to specify two parameters (alpha and beta). Since I used an elastic net predictor, alpha was set to 0.5. But the lambda value of was chosen by applying a 10 fold cross validation to the training data (via the R function cv.glmnet).

The elastic net regression results in a linear regression model whose coefficients b_0_, b_1_, . . . , relate to transformed age as follows
*F*(chronological age)=*b*_0_*+b*_1_*CpG*_1_*+ . . . +b*_p_*CpG*_p_+error

Note that the intercept term is denoted by b_0_. The coefficient values can be found in the attached Excel file.

Based, on the coefficient values from the regression model, DNAmAge is estimated as follows
*DNAm*Age=$F^{-1}$(*b*_0_*+b*_1_*CpG*_1_*+ . . . +b*_p_*CpG*_p_)

where $F^{-1}\left( y \right)$ denotes the mathematical inverse of the function F(.). Thus, the regression model can be used to predict to transformed age value by simply plugging the beta values of the selected CpGs into the formula.

**Defining Properties of the log linear transformation**

As indicated by its name, the “log-linear” function, has a logarithmic dependence on age before the average age of sexual maturity (of the species) and a linear dependence after Age at Sexual Maturity (of the species). For the human-Opossum clocks we used the following averages at sexual maturity (in units of years): 13.5 years for humans and 0.3343 years for Opossums.

Construction

We used a piecewise transformation, parameterized by Age of Sexual Maturity ($A$).

The transformation is F(x), given by

$$F\left( x \right)=g\left( \frac{x+1.5}{A+1.5} \right)\text{ where }g\left( t \right)= \left\{ \begin{aligned} \begin{aligned} \begin{aligned} \log\left( t \right), for 0\leq t\leq1 \\ t-1, for 1\leq t \end{aligned} \end{aligned} \end{aligned} \right.$$

Explicitly, F(x) is given by

$$F\left( x \right)=\left\{ \begin{aligned} \begin{aligned} \begin{aligned} \log\left( \frac{x+1.5}{A+1.5} \right), for 0\leq x\leq A \\ \frac{x-A}{A+1.5}, for A\leq x \end{aligned} \end{aligned} \end{aligned} \right.$$

In order to use this transformation to predict Age on *new samples*, one needs to use the *inverse* transformation, F^-1^(y), given by

$$F^{-1}\left( y \right)= \left\{ \begin{aligned} \begin{aligned} \begin{aligned} \left( A+1.5 \right)*\text{exp}\left( y \right)-1.5, for y\leq0 \\ (A+1.5)y+A, for y\geq0 \end{aligned} \end{aligned} \end{aligned} \right.$$

For predicting age, apply the inverse transformation to coefficient-weighted sum. That is,

$$DNAmAge=F^{-1}\left( x*\beta\right)$$

where $\beta$ is the vector of coefficients and $x$ is the vector of methylation values, with an intercept term.

**The DNAm Age estimate is estimated in two steps.**

First, one forms a weighted linear combination of the CpGs whose details can be found in Table

The table reports the probe identifier (cg number) used in the custom Infinium array (HorvathMammalMethylChip40). The weights used in this linear combination are specified in the respective column entitled "Coef.".

The formula assumes that the DNA methylation data measure "beta" values but the formula could be adapted to other ways of generating DNA methylation data.

**References**

[1] C. Y. McLean *et al.*, "GREAT improves functional interpretation of cis-regulatory regions," *Nat Biotechnol,* vol. 28, 2010// 2010, doi: 10.1038/nbt.1630.

[2] A. Arneson *et al.*, "A mammalian methylation array for profiling methylation levels at conserved sequences," *Nature Communications,* vol. 13, no. 1, p. 783, 2022/02/10 2022, doi: 10.1038/s41467-022-28355-z.

[3] S. Horvath, "DNA methylation age of human tissues and cell types," (in eng), *Genome Biol,* vol. 14, no. 10, p. R115, 2013, doi: 10.1186/gb-2013-14-10-r115.
